# 2,4-Bis(arylethynyl)-9-chloro-5,6,7,8-tetrahydroacridines: synthesis and photophysical properties

**DOI:** 10.3762/bjoc.17.115

**Published:** 2021-07-16

**Authors:** Najeh Tka, Mohamed Adnene Hadj Ayed, Mourad Ben Braiek, Mahjoub Jabli, Noureddine Chaaben, Kamel Alimi, Stefan Jopp, Peter Langer

**Affiliations:** 1 Asymmetric Synthesis and Molecular Engineering Laboratory for Organic Electronic Materials, Faculty of sciences of Monastir, Monastir university, Environment street, 5019 Monastir, Tunisia; 2 Universität Rostock, Institut für Chemie, Albert-Einstein-Str. 3a, 18059 Rostock, Germany; 3 Department of Chemistry, College of Science Al-zulfi, Majmaah University, Al-Majmaah, 11952, Saudi Arabia; 4 Université de Monastir, Faculté des Sciences, Unité de recherche sur les Hétéro-Epitaxies et Applications (URHEA), 5000 Monastir, Tunisia; 5 Leibniz-Institut für Katalyse e.V. an der Universität Rostock, Albert-Einstein-Str. 29a, 18059 Rostock, Germany

**Keywords:** alkynes, catalysis, cross-coupling, heterocycles, palladium

## Abstract

Acridine derivatives have attracted considerable interest in numerous areas owing to their attractive physical and chemical properties. Herein, starting from readily available anthranilic acid, an efficient synthesis of 2,4-bis(arylethynyl)-9-chloro-5,6,7,8-tetrahydroacridine derivatives was accomplished via a one-pot double Sonogashira cross-coupling method. The UV-visible absorption and emission properties of the synthesized molecules have been examined. Additionally, theoretical studies based on density functional theory (DFT/B3LYP/6-31G(d)) were carried out.

## Introduction

**Figure 1:**
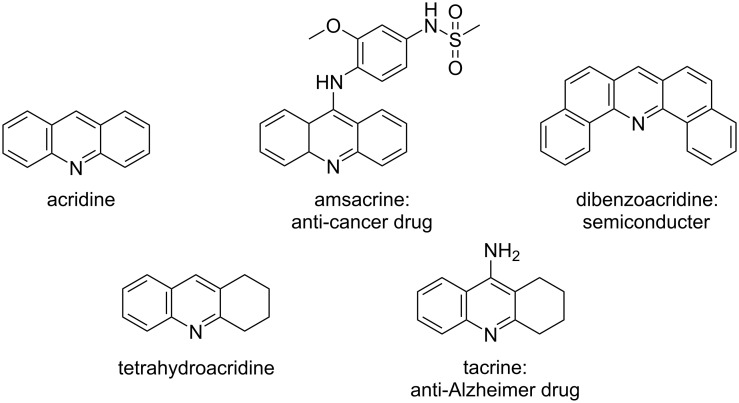
Applications of acridines.

The development and design of small π-conjugated molecules have attracted increasing attention for their inspiring applications in the fields of solar cells [1–3], organic devices [4–8], and as chemosensors [9–10]. The acridine core (Figure 1), formed by two benzenes fused to a pyridine ring, is among the most extensively studied heterocyclic aromatic compounds. It has first appeared as a side product during the synthesis of anthracene [11] and became an abundant scaffold in medicinal chemistry [12–14]. Acridine derivatives have exhibited a range of biological activities [15–20] and have been particularly explored in chemotherapeutic protocols against several types of tumors [21–31]_._ In recent years, much attention has been devoted to acridines in materials science due to their attractive photophysical and electrochemical properties [32–35]. They have been investigated in organic electronic devices [36–39] and were reported to be promising candidates for potential use as organic light emitting diodes [40]. Thus, various synthetic methodologies for the preparation of acridine-based molecules have been developed [41–47].

Tetrahydroacridine, containing a partially hydrogenated ring, is another privileged scaffold which showed interesting biological activities [48–54]. As a typical example, 9-amino-1,2,3,4-tetrahydroacridine or tacrine was the first drug approved for the treatment of Alzheimer's disease [55–57]. Surprisingly, photophysical properties of tetrahydroacridines have, to the best of our knowledge, not been studied so far. Recently, our research group reported the synthesis of a large variety of acridine derivatives which showed promising fluorescence properties and high quantum yields [58–60]. In continuation of our previous studies and as a part of our interest in discovering new organic materials applications [61–63], we herein report the synthesis of new 2,4-bis(arylethynyl)-9-chloro-5,6,7,8-tetrahydroacridine derivatives. The investigation of their photophysical properties and theoretical DFT studies were achieved aiming to understand the influence of substituents at introduced arylethynyl groups.

## Results and Discussion

### Synthesis

**Scheme 1:**
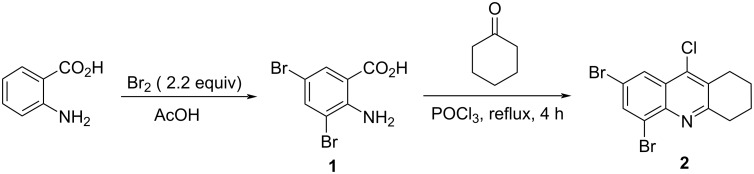
Synthesis of 2,4-dibromo-9-chloro-5,6,7,8-tetrahydroacridine (**2**).

At the outset of this study, we prepared 2,4-dibromo-9-chloro-5,6,7,8-tetrahydroacridine (**2**) following a two-step approach. We first prepared 3,5-dibromoanthranilic acid (**1**) by refluxing anthranilic acid with 2.2 equivalents of bromine in acetic acid as previously reported [64]. Subsequently, the POCl_3_-mediated cyclodehydration of **1** and cyclohexanone afforded **2** through an adapted reported procedure (Scheme 1) [65].

**Scheme 2:**
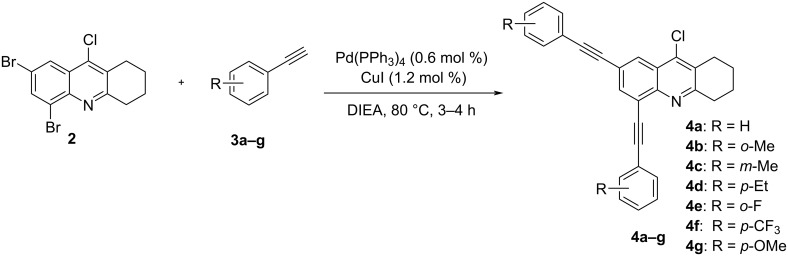
Synthesis of 2,4-bis(arylethynyl)-9-chloro-5,6,7,8-tetrahydroacridines **4a–g**.

Tetrahydroacridine **2** represents a novel synthetic building block for Pd catalysis. With this precursor in hand, we intended to expand the π-conjugation by introducing two arylethynyl groups by Sonogashira reactions [66–69]. For the optimization, we studied the reaction of **2** with phenylacetylene (**3a**) and we obtained the desired product **4a** in up to 72% as best yield using 0.6 mol % of tetrakis(triphenylphosphine)palladium(0) and 1.2 mol % of copper iodide (Scheme 2, Table 1).

**Table 1:** Effects of solvent, base, and temperature on the Sonogashira coupling between **2** and **3a**.^a^

Entry	Temperature	Base	Solvent	Yield^b^ (%)

1	80	Et_3_N	dioxane	72
2 3	80 80	Et_3_N Et_3_N	toluene DMF	70 68
4 5 6 7	80 80 90 100	iPr_2_EtN iPr_2_EtN iPr_2_EtN iPr_2_EtN	dioxane – – –	80 90 87 88

^a^Reagents and conditions: Pd(PPh_3_)_4_ (0.6 mol %), CuI (1.2 mol %), solvent (3 mL), base (0.5 mL), **2** (0.5 mmol), **3a** (1.1 mmol), 80 °C, 3 h. ^b^Isolated yield.

The reaction proceeded chemoselectively at the two carbon–bromine bonds giving 2,4-bis(phenylethynyl)-9-chloro-5,6,7,8-tetrahydroacridine (**4a**). This result was not entirely predictable, as the chlorine atom is located at the more reactive electron-poor pyridine moiety of the heterocyclic core structure. In fact, the chlorine position proved to be quite unreactive and all attempts to isolate 2,4,9-tris(phenylethynyl)-5,6,7,8-tetrahydroacridine failed even after using an excess of phenylacetylene and prolonging the reaction time. In order to study the regioselectivity of the reaction, a series of experiments were carried out with decreasing amounts of phenylacetylene. Although we used one equivalent of phenylacetylene, we were not able to isolate the mono coupling product.

Concerning the catalyst performance, Pd(PPh_3_)_4_ was found to be a suitable catalyst. In contrast, PdCl_2_(PPh_3)2_ was slightly less effective and gave lower yields. The replacement of dioxane by toluene or DMF did not lead to any significant improvement of the yields (Table 1, entries 2 and 3). For further improvement of the coupling, we evaluated the effect of the organic base. We found that the use of DIPEA instead of Et_3_N afforded better yields (Table 1, entry 4). Besides, the use of DIPEA as base and solvent gave a significant improvement of the yield (Table 1, entry 5). Our final effort consisted in evaluating the effect of the temperature. We found that increasing the temperature to 90 or 100 °C did not lead to any improvement (Table 1, entries 6 and 7).

The best result for the Sonogashira coupling reaction between intermediate **2** and phenylacetylene (**3a**) was obtained using 0.6 mol % of Pd(PPh_3_)_4_, 1.2 mol % of CuI in DIPEA at 80 °C for three hours. With the optimized conditions in hand, we examined the scope of the coupling reaction of **2** with different phenylacetylenes **3b–g**. As shown in Table 2, tetrahydroacridine derivatives **4a–g** were obtained in moderate to good yields. The yields were better for acetylenes containing electron-donating substituents. For example, arylacetylene **3g**, bearing a methoxy group, gave the best chemical yield of 93%. However, in case of the electron-attracting trifluoromethyl group (**3e**), we obtained a somewhat lower, but still good yield of 75%.

**Table 2:** Yields of 2,4-bis(arylethynyl)-9-chloro-5,6,7,8-tetrahydroacridine derivatives **4a–g**.

Entry	Arylacetylene	Product	Yield^a^ (%)	Time (h)

1	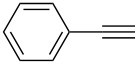	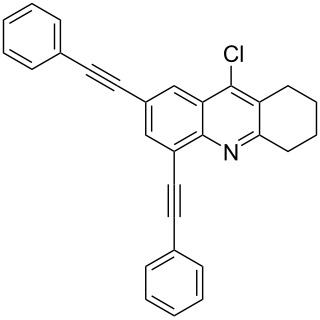 **4a**	90	3
2	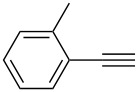	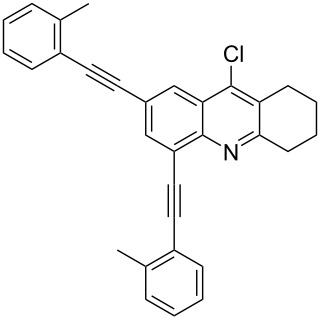 **4b**	85	3
3	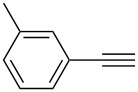	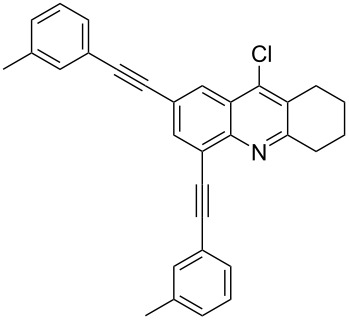 **4c**	82	3
4	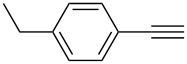	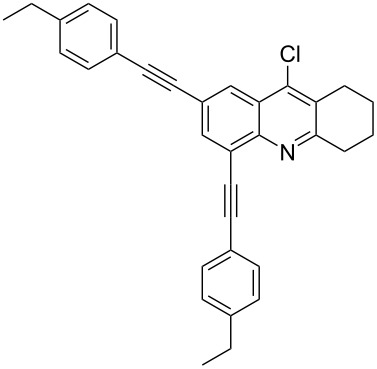 **4d**	83	3
5	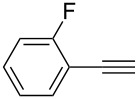	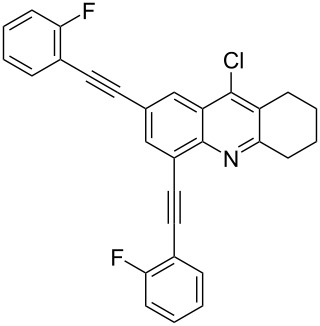 **4e**	80	4
6	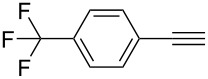	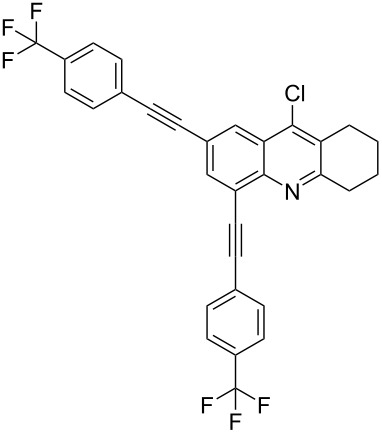 **4f**	75	4
7	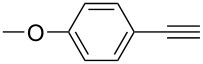	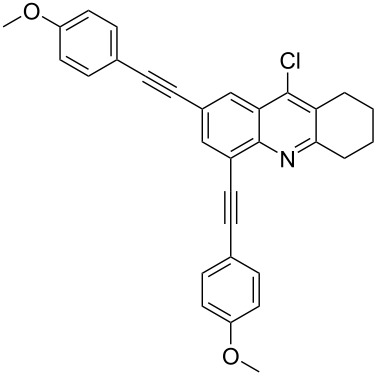 **4g**	93	4

^a^Isolated yields.

### Photophysical properties

**Figure 2:**
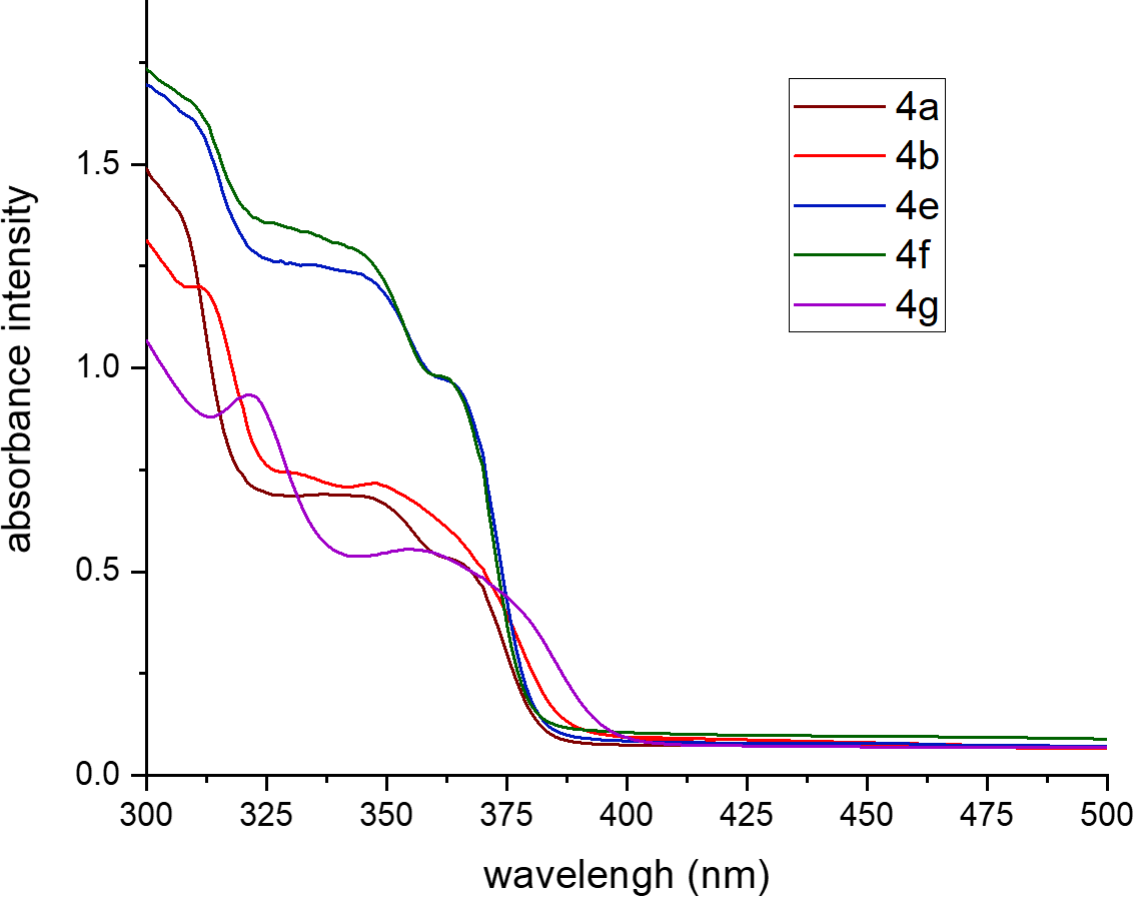
UV–vis absorption spectra of **4a**,**b** and **4e–g** in diluted dichloromethane solutions at room temperature (*c* = 1 × 10^−5^ M).

**Figure 3:**
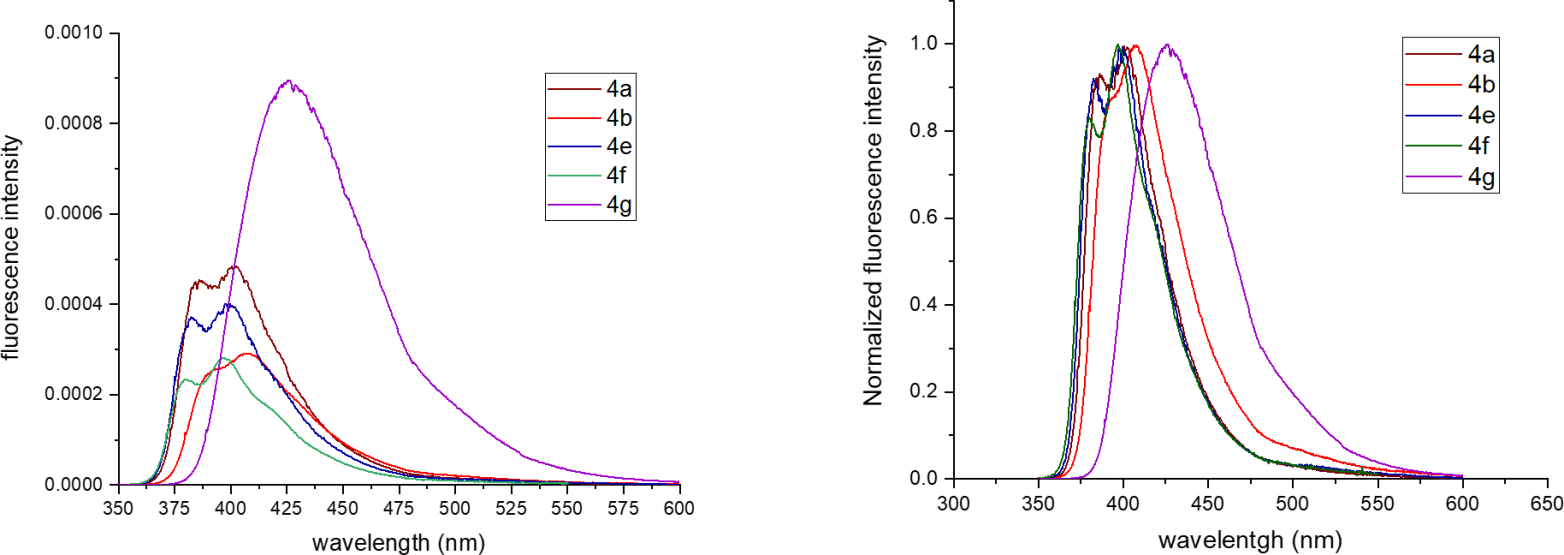
Emission spectra of **4a**,**b** and **4e–g** in diluted dichloromethane solutions at room temperature (*c* = 1 × 10^−5^ M).

As a prominent blue fluorescence was observed for the prepared tetrahydroacridine derivatives, their photophysical properties were investigated. Absorption and emission spectra were measured at room temperature in diluted dichloromethane solution and are depicted in Figure 2 and Figure 3. All spectroscopic data, including the maximum of absorption and emission, fluorescence quantum yield, stokes shift, onset of the absorption wavelengths and optical band gap are summarized in Table 3. Aiming to understand the impact of substituents at arylethynyl groups, spectra of diversely substituted tetrahydroacridines were compared with unsubstituted derivative **4a** taken as reference.

**Table 3:** Photophysical properties of **4a**,**b** and **4e–g** in dichloromethane solutions.

	Emission				Absorption			
	λ_em_ (nm)	FWHM^a^	Φ_fluo_^b^	ν̃ stokes^c^	λ_abs_ (nm )	log ε	λ_onset_ (nm)	*E*_g_^opt^ (eV)^d^

**4a**	386 400	48	0.11	2400	346 365	4.83 4.71	389	3.18
**4b**	394 406	55	0.10	4100	311 348	5.10 4.85	406	3.05
**4e**	382 398	50	0.14	2600	343 361	5.08 4.98	385	3.22
**4f**	380 396	50	0.11	2400	339 362	5.11 4.98	387	3.20
**4g**	426	66	0.20	4300	321 360	4.96 4.73	415	2.98

^a^Spectrum full width at half maximum. ^b^Fluorescence standard: quinine bisulfate in 1 N H_2_SO_4_ (Φ_fluo_= 0.54) [70]. ^c^Stokes shift in wavenumber (cm^−1^) = (1/λ_abs_^max^ − 1/λ_em_^max^ ) 10^7^. ^d^Estimated from the onset point of the absorption spectra: *E*_g_^opt^ = 1240/λ_onset_ [71].

The UV–vis absorption was measured in a spectral rang of 300 nm to 600 nm. The optical absorption spectra of all compounds spread over the UV range and showed wide absorption bands. These bands are assigned to a π→π* electronic transitions of the quinoline core and its two arylethynyl groups. As shown in Figure 2, the unsubstituted derivative **4a** exhibited wide bands with two maxima at 346 and 365 nm. A methyl group at the *ortho* position have a minor impact and derivative **4b** showed similar optical transitions with a slight red shift. While, derivatives **4e** and **4f** bearing an electron-deficient fluoro or trifluoromethyl group show a hyperchromic shift of their bands located between 340 and 380 nm. In case of the electron-donating methoxy substituent (**4g**), a bathochromic shift was observed. Besides, a new band appeared at 321 nm which may be attributed to intermolecular charge transfer between the oxygen lone pair electrons and the quinoline core. The unsubstituted derivative **4a** presents an onset of absorption (λ_onset_) at 389 nm and its optical band gap was deduced to be around 3.18 eV. Tetrahydroacridines **4e** and **4f** showed approximately the same optical band gaps. However, the optical band gaps of **4b** and **4g** are lower (3.05 eV and 2.98 eV, respectively).

Emission spectra of synthesized tetrahydroacridine derivatives were measured in dichloromethane solutions under UV-laser excitation of 325 nm. The emission spectrum of compound **4a** presents a profile with two transitions located at 386 and 400 nm. Methyl-substituted derivative **4b** gave a slight red shift of 10 nm as compared to **4a**. In contrast, fluorine and trifluoromethyl-substituted derivatives **4e** and **4f** show nearly the same emission. However, derivative **4g** containing an electron-donating methoxy substituent exhibits a larger red shift of around 40 nm. Based on the absorption and emission spectra, the prepared tetrahydroacridine derivatives possess stokes shifts (wavenumber) ranging from 2400 to 4300 cm^−1^. Their fluorescence quantum yields range from 0.1 to 0.2 as measured according to a relative method using quinine sulfate [70]. Tetrahydroacridine derivative **4g** containing an electron-donating methoxy substituent gave the highest fluorescence intensity as shown in Figure 3 and a quantum yield of 20%.

### DFT studies

The arylethynyl substituents showed an impact on the absorption and emission. In order to elucidate these experimental observations, quantum chemical calculations based on density functional theory (DFT) methodology were performed. The estimated visualization of highest occupied and lowest unoccupied molecular orbitals, as well as the molecular electrostatic potential (MEP) of prepared products are given in Table 4.

**Table 4:** Visualization of HOMO, LUMO orbitals and MEP.

	HOMO	LUMO	MEP

**4a**	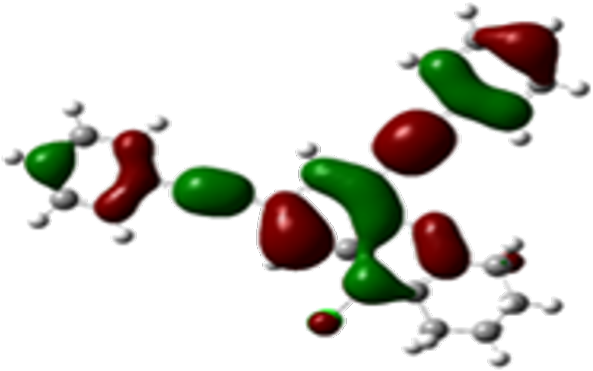	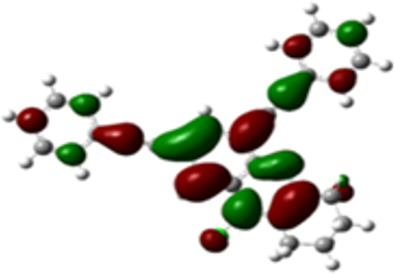	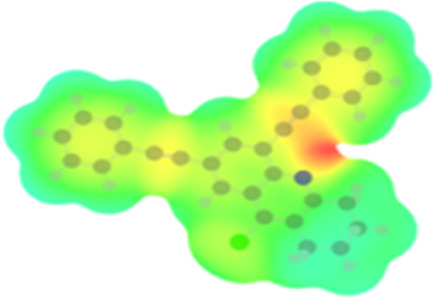
**4b**	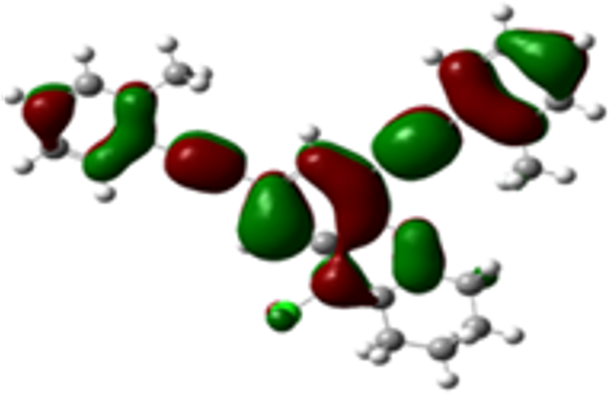	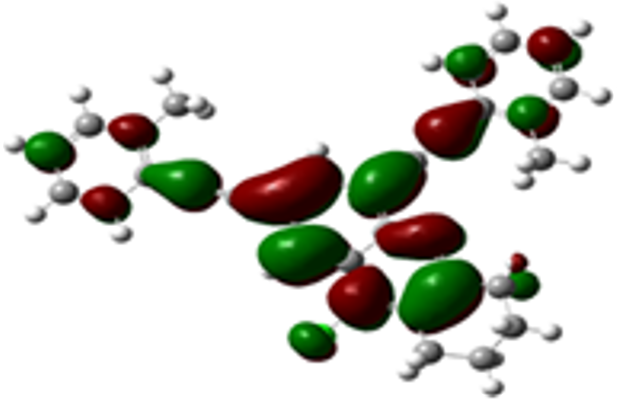	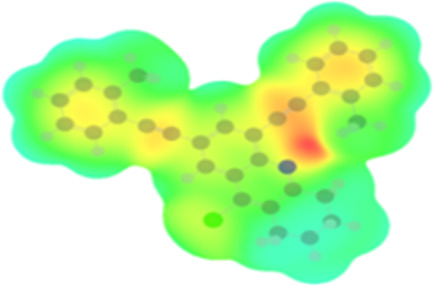
**4e**	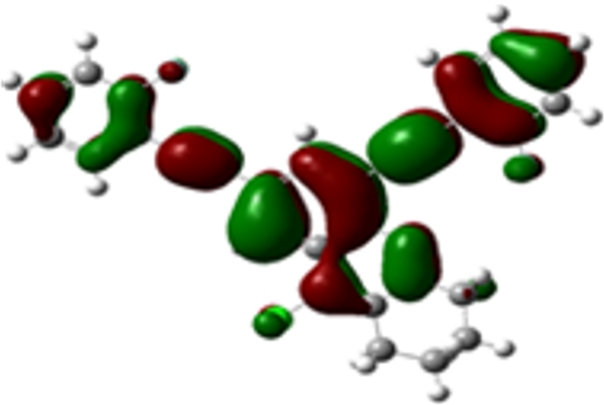	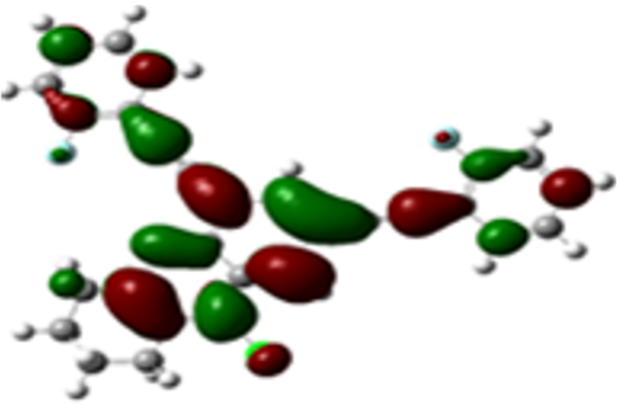	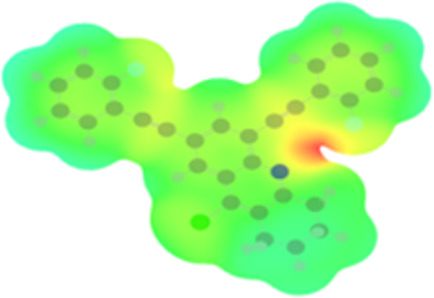
**4f**	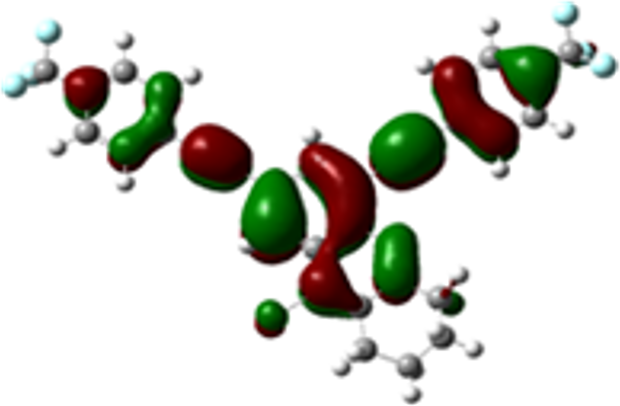	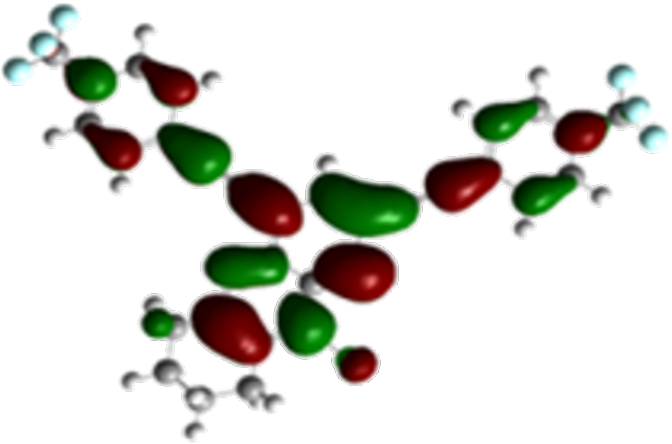	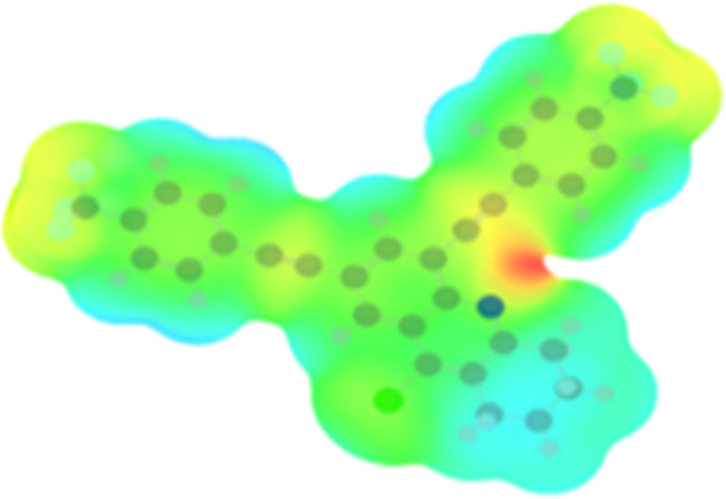
**4g**	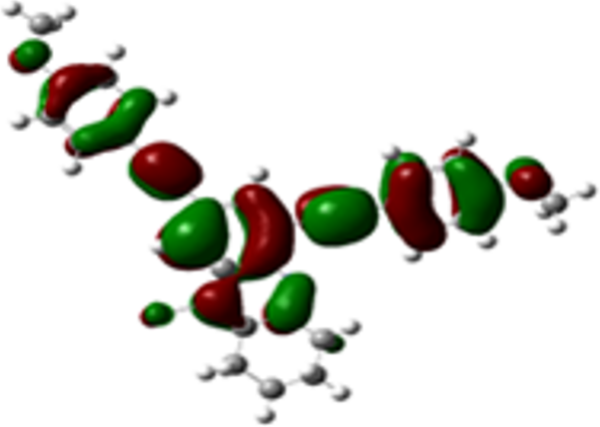	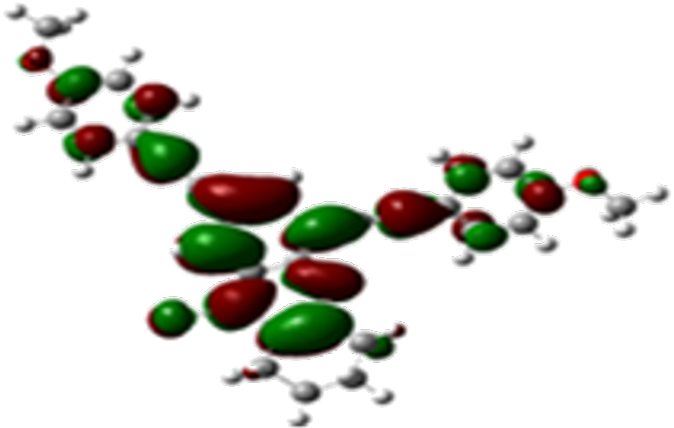	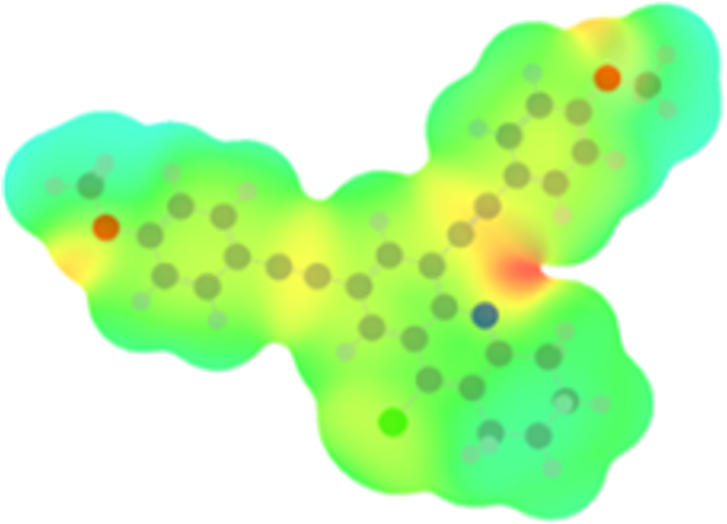

For phenylethynyl-substituted product **4a**, the blue colored surface, located mainly at the cyclohexane ring, visualizes the electron deficiency. While the red region, localized essentially at the nitrogen atom and its closer ethynyl group, show the electron abundance. Due to their low donating effect, the methyl group in product **4b** induce an addition of yellow regions into the external phenyl rings. However, the electron-deficient fluorine atom in derivative **4e** results in a decrease of the electron density of the tetrahydroacridine core and the external phenyl rings. For product **4f**, the high electron-deficient effect of the trifluoromethyl groups induces the appearance of blue surfaces around the tetrahydroacridine core, the ethynyl groups and the external phenyl rings, indicating a significant decrease of their electronic densities. However, a yellow-red region is added to the electrostatic map of compound **4g**, due to the positive mesomeric effect of the π-donating methoxy substituent. Accordingly, we conclude that substituents at the introduced arylethynyl groups can communicate electronically with the central tetrahydroacridine core via the ethynyl group. Consequently, they influence the electronic situation of the prepared tetrahydroacridines and are expected to change their structural proprieties. Hence, some structural parameters including gap (*E*_g_), ionization potential (IP), electron affinity (EA) and dipole moments (µ) were deduced on the ground state from the optimized chemical structure of obtained molecules (Table 5).

**Table 5:** Summary of theoretical calculations.^a^

Compound	*E*_HOMO_	*E*_LUMO_	IP	EA	*E*_g_ = *E*_LUMO_ - E_HOMO_ (eV)	μ (D)

**4a**	−5.499	−1.992	5.5	1.99	3.5	0.698
**4b**	−5.465	−2.00	5.26	2	3.26	0.970
**4e**	−5.553	−2.033	5.55	2.0	3.55	0.657
**4f**	−5.887	−2.356	5.887	2.35	3.53	4.276
**4g**	−5.055	−1.742	5.178	1.8	3.36	1.669

^a^The DFT calculations were performed on optimized geometries with a DFT/b3lyp/6-31g(d).

The calculated permanent dipole moments µ (D) have considerably increased values for **4f** and **4g**, which show significant changes in their experimental emission properties. The presence of six fluorine atoms induces a large polarity difference. In fact, derivative **4f** shows the highest dipole moment of 4.276 D as compared to **4a** (0.698 D). As shown in Table 5, the ionization potential (IP) and electron affinity (EA) of tetrahydroacridines are almost identical. In addition, *E*_HOMO_ and *E*_LUMO_ do not change notably and the calculated *E*_g_ values vary only slightly from 3.26 to 3.55 eV. Although, the HOMO energy level of **4g** with −5.055 eV is higher than −5.887 eV of **4e**, both compounds have close band gap values of 3.36 eV and 3.53 eV, respectively.

## Conclusion

In summary, we have reported a facile synthesis of 2,4-bis(arylethynyl)-9-chloro-5,6,7,8-tetrahydroacridine derivatives via a double Sonogashira cross-coupling method. The arylethynyl groups expand the π-conjugation of the tetrahydroacridine core. The substituents located at the aryl group influenced the photophysical properties of the prepared molecules. In particular, the methoxy derivative shows promising fluorescence properties.

## Experimental

### Materials and measurements

All reactions were carried out under an inert argon atmosphere. Anhydrous solvents and chemicals were purchased from Sigma-Aldrich and used without further purification. All reactions were monitored by thin-layer chromatography (TLC) using commercial silica-gel plate 60 coated with a fluorescence indicator and the visualization was performed by UV (254 nm). Organic compounds were purified using Merck Silica gel 60 (0.043–0.06 mm). Solvents for work-up and column chromatography were distilled before use.

NMR data were recorded on Bruker ARX 300 instruments in CDCl_3_ with tetramethylsilane as the internal standard (signals due to the solvent; CHCl_3_: δ 7.26 for ^1^H and δ 77.16 for ^13^C). The ^1^H NMR chemical shifts and coupling constants were determined assuming first-order behavior. Peak characterization of ^1^H NMR spectra: s = singlet, d = doublet, t = triplet, q = quartet, m = multiplet. Chemical shifts were given in ppm (δ) relative to tetramethylsilane (SiMe_4_). Photophysical studies were carried out in freshly prepared dichloromethane solutions with concentrations of 1 × 10^−5^ M. UV–vis spectra were recorded on a Shimadzu 2401 PC spectrophotometer in quartz cuvettes with a path length of 1 cm. Emission spectra were recorded on a Perkin-Elmer LS50B spectrofluorimeter.

### Theoretical calculations

Theoretical studies were realized in vacuum with Gaussian 09 program [72]. The geometry of the equilibrium conformer at ground state was first found at AM1 level. Then, further optimizations through density functional theory (DFT) approach [73] at the restricted Becke3–Lee–Yang–Parr hybrid functional (B3LYP) with standard basis set 6-31G were carried out.

### Experimental procedure and spectroscopic data for 2,4-dibromo-9-chloro-5,6,7,8-tetrahydroacridine (**2**)

3,5-Dibromoanthranilic acid (2.92 g, 10 mmol, 1 equiv) and cyclohexanone (1.07 mL, 11 mmol, 1.1 equiv) were stirred in an ice bath. Then, 15 mL of POCl_3_ was carefully added and the mixture was heated under reflux for 4 hours. The mixture was cooled to room temperature and concentrated to give a slurry. The residue was diluted with dichloromethane, neutralized with aqueous NaHCO_3_, and washed with brine. The organic layer was dried over anhydrous K_2_CO_3_ and concentrated to afford a yellow solid. It was recrystallized from acetone to give **2** as pale yellow solid (3.24 g, 87%) [65]. ^1^H NMR (300 MHz, CDCl_3_) δ 1.85–1.95 (m, 4H, CH_2_-CH_2_), 2.93 (t, ^3^*J* = 6.0 Hz, 2H, CH_2_), 3.11 (t,^3^*J* = 6.0 Hz, 2H, CH_2_), 8.05 (s, 1H, aryl-H), 8.21 (s, 1H, aryl-H); ^13^C NMR (75 MHz, CDCl_3_) δ 22.40 (CH_2_), 22.42 (CH_2_), 27.63 (CH_2_), 34.45 (CH_2_), 119.73 (C_Ar_), 125.37 (C_Ar_), 126.00 (C_Ar_), 127.16 (C_Ar_), 130.78 (C_Ar_), 135.58 (Cl-C_Ar_), 140.36 (C_Ar_), 142.46 (C_Ar_), 161.25 (N =C_Ar_).

### Experimental procedure for the Sonogashira coupling and spectroscopic data for 2,4-bis(arylethynyl)-9-chloro-5,6,7,8-tetrahydroacridine derivatives **4a–g**

2,4-Dibromo-9-chloro-5,6,7,8-tetrahydroacridine (**2**, 372.89 mg, 1 mmol, 1 equiv), arylacetylene (2.2 mmol, 2.2 equiv), Pd(PPh_3_)_4_ (6.9 mg, 0.006 mmol, 0.6 mol %) and CuI (2.2 mg, 0.012 mmol, 1.2 mol %) were added to a dried glass pressure tube. The tube was evacuated and backfilled three times with argon, then diisopropylethylamine (4.0 mL) was added. The tube was sealed with a Teflon cap and heated to 80 °C for 3–4 hours until completion of the reaction (monitored by TLC). The mixture was then cooled to room temperature and the solvent was removed under reduced pressure. Water (10 mL) was added and the solution was extracted using CH_2_Cl_2_ (3 × 10 mL). The combined organic layers were dried (Na_2_SO_4_) and the solvent was evaporated under reduced pressure. The crude product was purified by column chromatography using heptanes/ethyl acetate 9:1 as eluent.

**2,4-Bis(phenylethynyl)-9-chloro-5,6,7,8-tetrahydroacridine (4a):** colorless solid (375 mg, 0.9 mmol, 90%); mp 188–190 °C; ^1^H NMR (300 MHz, CDCl_3_) δ 1.81–1.92 (m, 4H, CH_2_-CH_2_), 2.94 (t, ^3^*J* = 6.0 Hz, 2H, CH_2_), 3.21 (t, ^3^*J* = 6.0 Hz, 2H, CH_2_), 7.29–7.35 (m, 6H, aryl-H), 7.49–7.53 (m, 2H, aryl-H), 7.64–7.68 (m, 2H, aryl-H), 8.01 (s, 1H, aryl-H), 8.24 (s, 1H, aryl-H); ^13^C NMR (75 MHz, CDCl_3_) δ 22.4 (CH_2_), 22.5 (CH_2_), 30.9 (CH_2_), 34.4 (CH_2_), 86.5 (C_sp_), 88.5 (C_sp_), 91.1 (C_sp_), 96.2 (C_sp_), 121.3 (C_Ar_), 122.8 (C_Ar_), 123.0 (C_Ar_), 123.3 (C_Ar_), 125.4 (C_Ar_), 127.2 (C_Ar_), 128.3 (C_Ar_), 128.4 (C_Ar_), 128.5 (C_Ar_), 128.6 (C_Ar_), 130.2 (C_Ar_), 131.7 (C_Ar_), 132.0 (C_Ar_), 136.2 (Cl-C_Ar_), 141.5 (C_Ar_), 145.4 (N-C_Ar_), 161.1 (N =C_Ar_); HRMS (ESI): [M]^+^ calcd for C_29_H_20_ClN, 417.1284; found, 417.1265.

**2,4-Bis(*o*-tolylethynyl)-9-chloro-5,6,7,8-tetrahydroacridine (4b):** pale green solid (385 mg, 0.85 mmol, 85%); mp 119–120 °C; ^1^H NMR (300 MHz, CDCl_3_) δ 1.78–1.93 (m, 4H, 2CH_2_), 2.52 (s, 3H, aryl-CH_3_), 2.66 (s, 3H, aryl-CH_3_), 2.92 (t, ^3^*J* = 6.0 Hz, 2H, CH_2_), 3.24 (t, ^3^*J* = 6.0 Hz, 2H, CH_2_), 7.12–7.25 (m, 6H, aryl-H), 7.42–7.51 (m, 1H, aryl-H), 7.61–7.66 (m, 1H, aryl-H), 7.95 (s, 1H, aryl-H), 8.22 (s, 1H, aryl-H); ^13^C NMR (75 MHz, CDCl_3_) δ 20.8 (CH_3_), 20.9 (CH_3_), 22.4 (CH_2_), 22.4 (CH_2_), 27.5 (CH_2_), 34.1 (CH_2_), 90.1 (C_sp_), 90.2 (C_sp_), 92.3 (C_sp_), 95.8 (C_sp_), 119.7 (C_Ar_), 121.80 (C_Ar_), 122.9 (C_Ar_), 123.1 (C_Ar_), 125.5 (C_Ar_), 126.8 (C_Ar_), 128.6 (C_Ar_), 128.8 (C_Ar_), 129.4 (C_Ar_), 129.6 (C_Ar_), 130.3 (C_Ar_), 130.4 (C_Ar_), 132.1 (C_Ar_), 135.8 (Cl-C_Ar_), 141.1 (C_Ar_), 145.0 (N-C_Ar_), 160.8 (N =C_Ar_); HRMS (ESI): [M]^+^ calcd for C_31_H_24_ClN, 445.1597; found, 445.1576.

**2,4-Bis(*m*-tolylethynyl)-9-chloro-5,6,7,8-tetrahydroacridine (4c):** pale black solid (364 mg, 0.82 mmol, 82%); mp 170–172 °C; ^1^H NMR (300 MHz, CDCl_3_) δ 1.80–1.94 (m, 4H, 2CH_2_), 2.31 (s, 3H, aryl-CH_3_), 2.33 (s, 3H, aryl-CH_3_), 2.92 (t, ^3^*J* = 6.0 Hz, 2H, CH_2_), 3.21 (t, ^3^*J* = 6.0 Hz, 2H, CH_2_), 7.05–7.36 (m, 4H, aryl-H), 7.40–7.51 (m, 2H, aryl-H), 7.58–7.64 (m, 2H, aryl-H), 7.91 (s, 1H, aryl-H), 8.21 (s, 1H, aryl-H); ^13^C NMR (75 MHz, CDCl_3_)δ 21.27 (CH_3_), 21.29 (CH_3_), 22.41 (CH_2_), 22.49 (CH_2_), 27.56 (CH_2_), 34.36 (CH_2_), 86.10 (C_sp_), 88.17 (C_sp_), 91.42 (C_sp_), 96.58 (C_sp_), 121.53 (C_Ar_), 122.58 (C_Ar_), 122.96 (C_Ar_), 123.15 (C_Ar_), 125.51 (C_Ar_), 127.08 (C_Ar_), 128.19 (C_Ar_), 128.36 (C_Ar_), 129.15 (C_Ar_), 129.62 (C_Ar_), 130.25 (C_Ar_), 132.60 (C_Ar_), 136.37 (C_Ar_), 138.16 (Cl-C_Ar_), 141.79 (C_Ar_), 145.15 (N-C_Ar_), 160.99 (N =C_Ar_); HRMS (ESI): [M]^+^ calcd for C_31_H_24_ClN, 445.1597; found, 445.1584.

**2,4-Bis((*p*-ethylphenyl)ethynyl)-9-chloro-5,6,7,8-tetrahydroacridine (4d):** pale black solid (392 mg, 0.83 mmol, 83%); mp 189–191 °C; ^1^H NMR (300 MHz, CDCl_3_) δ 1.19 (t, 6H, 2CH_3_), 1.82–1.93 (m, 4H, 2CH_2_), 2.54 (q, 4H, 2CH_2_), 2.93 (t, ^3^*J* = 6.0 Hz, 2H, CH_2_), 3.28 (t, ^3^*J* = 6.0 Hz, 2H, CH_2_), 7.11–7.19 (m, 4H, aryl-H), 7.35–7.42 (m, 2H, aryl-H), 7.62–7.69 (m, 2H, aryl-H), 7.96 (s, 1H, aryl-H), 8.23 (s, 1H, aryl-H); ^13^C NMR (75 MHz, CDCl_3_) δ 15.31 (CH_3_), 15.32 (CH_3_), 22.26 (CH_2_), 22.37 (CH_2_), 27.56 (CH_2_), 28.89 (CH_2_), 28.91 (CH_2_), 33.99 (CH_2_), 85.45 (C_sp_), 87.75 (C_sp_), 91.77. (C_sp_), 97.24 (C_sp_), 119.84 (C_Ar_), 120.39 (C_Ar_), 122.03 (C_Ar_), 122.62 (C_Ar_), 125.60 (C_Ar_), 126.73 (C_Ar_), 127.86 (C_Ar_), 128.05 (C_Ar_), 130.41 (C_Ar_), 131.78 (C_Ar_), 132.14 (C_Ar_), 136.72 (Cl-C_Ar_), 142.60 (C_Ar_), 144.24 (C_Ar_), 145.08 (C_Ar_), 145.30 (N-C_Ar_), 160.84 (N=C_Ar_); HRMS (ESI): [M]^+^ calcd for C_33_H_28_ClN, 473.1910; found, 473.1924.

**2,4-Bis((*o*-fluorophenyl)ethynyl)-9-chloro-5,6,7,8-tetrahydroacridine (4e):** pale red solid (375 mg, 0.8 mmol, 80%); mp 179–181 °C; ^1^H NMR (300 MHz, CDCl_3_) δ 1.81–1.96 (m, 4H, 2CH_2_), 2.93 (t, ^3^*J* = 6.0 Hz, 2H, CH_2_), 3.25 (t, ^3^*J* = 6.0 Hz, 2H, CH_2_), 7.06–7.15 (m, 4H, aryl-H), 7.21–7.31 (m, 2H, aryl-H), 7.45–7.53 (m, 1H, aryl-H), 7.60–7.65 (m, 1H, aryl-H), 8.03 (s, 1H, aryl-H), 8.27 (s, 1H, aryl-H); ^13^C NMR (75 MHz, CDCl_3_) δ 21.3 (CH_2_), 21.4 (CH_2_), 26.5 (CH_2_), 33.4 (CH_2_), 83.5 (C_sp_), 88.6 (C_sp_), 90.3. (C_sp_), 92.3 (C_sp_), 110.3 (C_Ar_), 110.9 (C_Ar_), 114.3 (C_Ar_), 114.8 (C_Ar_), 120.0 (C_Ar_), 121.6 (C_Ar_), 122.9 (C_Ar_), 123.0 (C_Ar_), 124.4 (C_Ar_), 126.7 (C_Ar_), 129.2 (C_Ar_), 128.8 (C_Ar_), 129.4 (C_Ar_), 129.6 (C_Ar_), 132.9 (C_Ar_), 135.3 (Cl-C_Ar_), 140.8 (C_Ar_), 144.3 (N-C_Ar_), 159.8 (F-C_Ar_), 160.04 (F-C_Ar_). 163.8 (N=C_Ar_); HRMS (ESI): [M]^+^ calcd for C_29_H_18_ClF_2_N, 453.1095; found, 453.1081.

**2,4-Bis((*p*-trifluoromethylphenyl)ethynyl)-9-chloro-5,6,7,8-tetrahydroacridine (4f):** brown solid (414 mg, 0.75 mmol, 75%); mp 170–172 °C; ^1^H NMR (300 MHz, CDCl_3_) δ 1.81–1.95 (m, 4H, 2CH_2_), 2.95 (t, ^3^*J* = 6.0 Hz, 2H, CH_2_), 3.23 (t, ^3^*J* = 6.0 Hz, 2H, CH_2_), 7.51–7.75 (m, 8H, aryl-H), 8.05 (s, 1H, aryl-H), 8.31 (s, 1H, aryl-H); ^13^C NMR (75 MHz, CDCl_3_) δ 22.3 (CH_2_), 22.4 (CH_2_), 27.6 (CH_2_), 34.5 (CH_2_), 86.5 (C_sp_), 89.7 (C_sp_), 90.5 (C_sp_), 93.2 (C_sp_), 120.6 (C_Ar_), 122.4 (C_Ar_), 125.2 (F_3_C-C_Ar_), 125.3 (F_3_C-C_Ar_), 125.4 (C_Ar_), 125.5 (C_Ar_), 126.5 (C_Ar_), 127.1 (C_Ar_), 128.2 (C_Ar_), 129.9 (C_Ar_), 130.4 (C_Ar_), 130.6 (C_Ar_), 131.9 (C_Ar_), 132.2 (C_Ar_), 136.4 (Cl-C_Ar_), 141.8 (C_Ar_), 145.5 (N-C_Ar_), 161.7 (N =C_Ar_); HRMS (ESI): [M]^+^ calcd for C_31_H_18_ClF_6_N, 553.1032; found, 553.1015.

**2,4-Bis((*p*-methoxyphenyl)ethynyl)-9-chloro-5,6,7,8-tetrahydroacridine (4g):** yellow solid (536 mg, 0.93 mmol, 93%); mp 125–127 °C; NMR (300 MHz, CDCl_3_) δ 1.83–1.92 (m, 4H, 2CH_2_), 2.94 (t, ^3^*J* = 6.0 Hz, 2H, CH_2_), 3.33 (t, ^3^*J* = 6.0 Hz, 2H, CH_2_), 3.79 (s, 6H, 2 OCH_3_), 7.75–7.89 (m, 4H, aryl-H), 7.47 (d, 2H, aryl-H), 8.62 (d, 2H, aryl-H), 7.93 (s, 1H, aryl-H), 8.21 (s, 1H, aryl-H); ^13^C NMR (75 MHz, CDCl_3_) δ 22.2 (CH_2_), 22.3 (CH_2_), 27.5 (CH_2_), 33.9 (CH_2_), 55.3 (OCH_3_), 55.3 (OCH_3_), 87.2 (C_sp_), 88.7 (C_sp_), 90.2 (C_sp_), 92.2 (C_sp_), 113.9 (C_Ar_), 114.1 (C_Ar_), 114.7 (C_Ar_), 115.3 (C_Ar_), 122.2 (C_Ar_), 122.6 (C_Ar_), 125.6 (C_Ar_), 126.3 (C_Ar_), 130.0 (C_Ar_), 130.4 (C_Ar_), 132.9 (C_Ar_), 133.3 (C_Ar_), 133.7 (C_Ar_), 136.5 (Cl-C_Ar_), 143.1 (N-C_Ar_), 159.9 (OCH_3_-C_Ar_), 160.0 (OCH_3_-C_Ar_), 160.9 (N =C_Ar_); HRMS (ESI): [M]^+^ calcd for C_31_H_18_ClF_6_N, 577.1495; found, 577.1479.

## Supporting Information

File 1 Additional experimental data.
