# Supplementary material for: 2,4-Bis(arylethynyl)-9-chloro-5,6,7,8-tetrahydroacridines: synthesis and photophysical properties
Source: Beilstein J Org Chem. 2021 Jul 16;17:1629–40. doi: 10.3762/bjoc.17.115 (PMC8290096; doi:10.3762/bjoc.17.115)
Supplement: File 1 — Additional experimental data. [file Beilstein_J_Org_Chem-17-1629-s001.pdf]

BEILSTEIN JOURNAL OF ORGANIC CHEMISTRY

Supporting Information

for

2,4-Bis(arylethynyl)-9-chloro-5,6,7,8-tetrahydroacridines: synthesis and photophysical properties

Najeh Tka, Mohamed Adnene Hadj Ayed, Mourad Ben Braiek, Mahjoub Jabli, Noureddine Chaaben, Kamel Alimi, Stefan Jopp and Peter Langer

Beilstein J. Org. Chem. **2021,** *17,* 1629–1640. doi:10.3762/bjoc.17.115

Additional experimental data

License and Terms: This is a supporting information file under the terms of the Creative Commons Attribution License (https://creativecommons.org/licenses/by/4.0). Please note that the reuse, redistribution and reproduction in particular requires that the author(s) and source are credited and that individual graphics may be subject to special legal provisions.
The license is subject to the *Beilstein Journal of Organic Chemistry* terms and conditions: (https://www.beilstein-journals.org/bjoc/terms)

1. General comments

All reactions were carried out under an inert argon atmosphere. Anhydrous solvents and chemicals were purchased from Sigma-Aldrich and used without further purification. All reactions were monitored by thin-layer chromatography (TLC) using commercial silica-gel plate 60 coated with a fluorescence indicator and the visualization was performed by UV (254 nm). Organic compounds were purified using Merck Silica gel 60 (0.043–0.06 mm). Solvents for work-up and column chromatography were distilled before use. NMR data were recorded on Bruker ARX 300 instruments in $CDCl_3$ with tetramethylsilane as the internal standard (Signals due to the solvent; $CHCl_3$: δ 7.26 for ^{1}H and δ 77.16 for ^{13}C). The ^{1}H NMR chemical shifts and coupling constants were determined assuming first-order behavior. Peak characterization of ^{1}H NMR spectra: s = singlet, d = doublet, t = triplet, q = quartet, m = multiplet. Chemical shifts were given in ppm (δ) relative to tetramethylsilane $SiMe_4$ as internal standard. Mass spectrometry (MS) was carried out on Finnigan MAT 95 XP (electron ionization EI,70 eV); 6890 N/5973 (Agilent), 6210 Time-of-Flight LC/MS (Agilent); Gas Chromatography MS (GC–MS): Agilent HP- 5890 with an Agilent HP-5973 Mass Selective Detector (EI) and HP-5 capillary column using helium carrier gas. The High resolution MS [HR-MS (ESI)] was performed on Agilent 1969 A TOF.

2. Quantum yield calculation:

We have calculated Quantum yield with respect to quinine sulphate (SQ) in 0.1 M H_2SO_4, using the following formula:

$$\Phi = \Phi_{ST}\left[\frac{Grad}{Grad_{ST}}\right]\left[\frac{\eta^2}{\eta^2_{ST}}\right]$$

Where, Φ: quantum yield of sample; Φ_{ST}: quantum yield of quinine sulphate (0.54); Grad: the slope of the plot of integrated fluorescence intensity vs absorbance of sample; $Grad_{ST}$: the slope

of the plot of integrated fluorescence intensity vs absorbance of quinine sulphate; η : the refractive index of dichloromethane (1.42); η_{ST} : the refractive index of water (1.33).

As example, the fluorescence quantum yield (Φ) value of tetrahydroacridine derivative **4g** was 20 % as calculated follows:

Φ (4g) = 0.54 * (0.053/0.157) * $(1.42/1.33)^2$ = 0.20

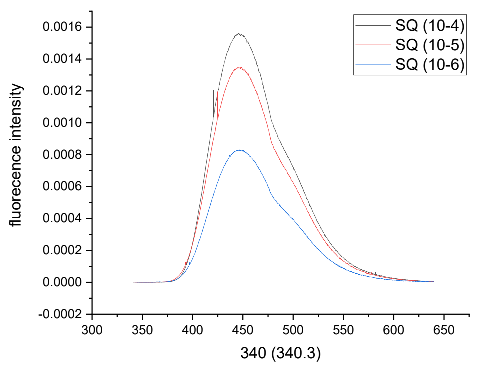

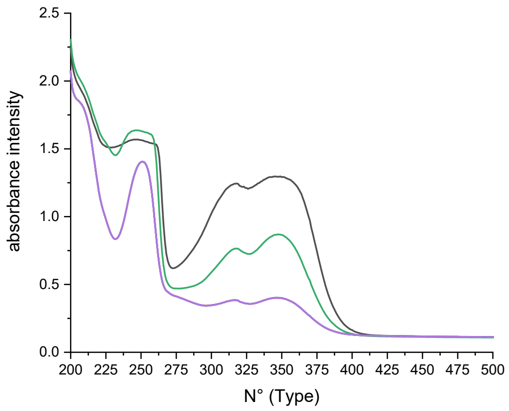

Fluorescence and absorbance spectra for the standard quinine sulfate.

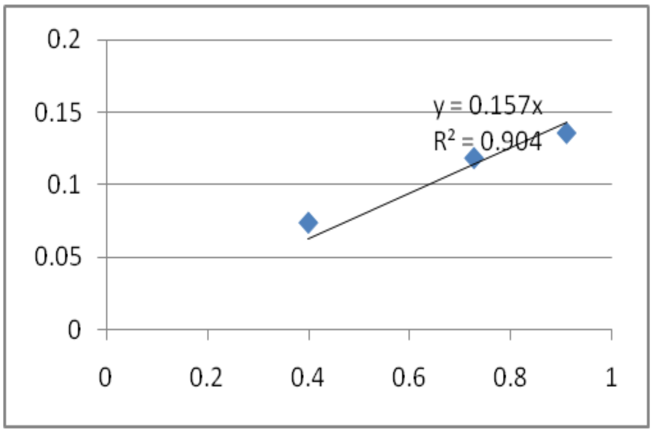

Plot of fluorescence intensities against their absorbances for the standard quinine sulfate.

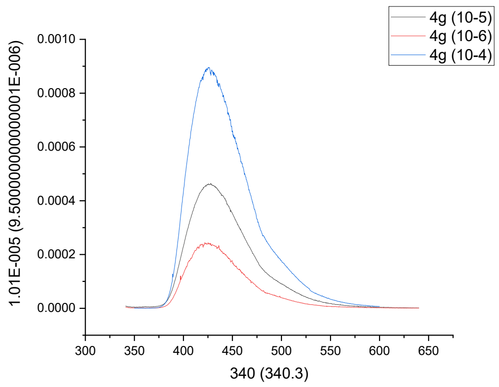

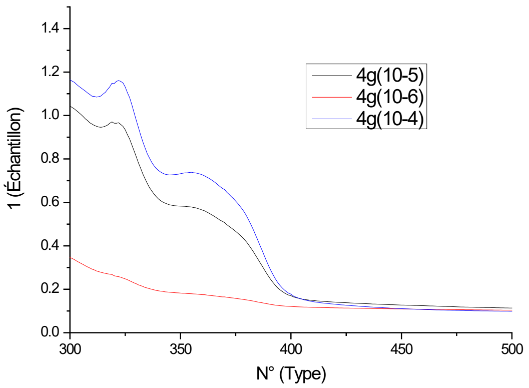

Fluorescence and absorbance spectra for 4g

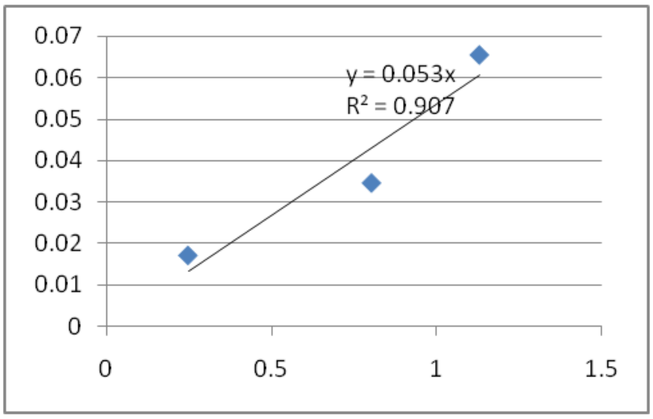

Plot of fluorescence intensities against their absorbances for 4g

3. Procedure for preparing the starting material 2,4-dibromo-9-chloro-5,6,7,8-tetrahydroacridine (2):

3,5-Dibromoanthranilic acid (2.92 g, 10 mmol, 1 equiv) and cyclohexanone (1.07 mL, 11 mmol, 1.1 equiv) were stirred in an ice bath. Then, 15 mL of $POCl_3$ was carefully added and the mixture was heated under reflux for 4 hours. The mixture was cooled at room temperature and concentrated to give a slurry. The residue was diluted with dichloromethane, neutralized with aqueous $NaHCO_3$, and washed with brine. The organic layer was dried over anhydrous K_2CO_3 and concentrated to afford a yellow solid. It was recrystallized from acetone to give **2** as pale yellow solid (3.24 g, 87%).

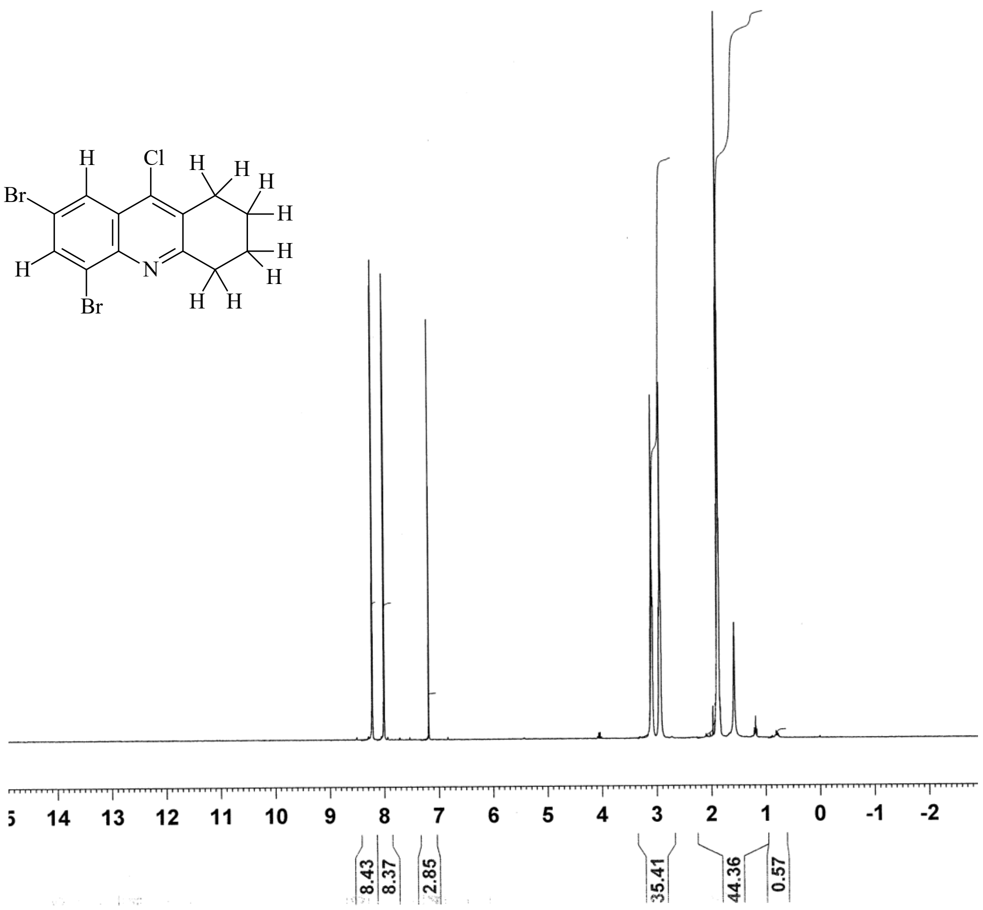

^{1}H NMR (300 MHz, CDCl3) of 2,4-dibromo-9-chloro-5,6,7,8-tetrahydroacridine (2)

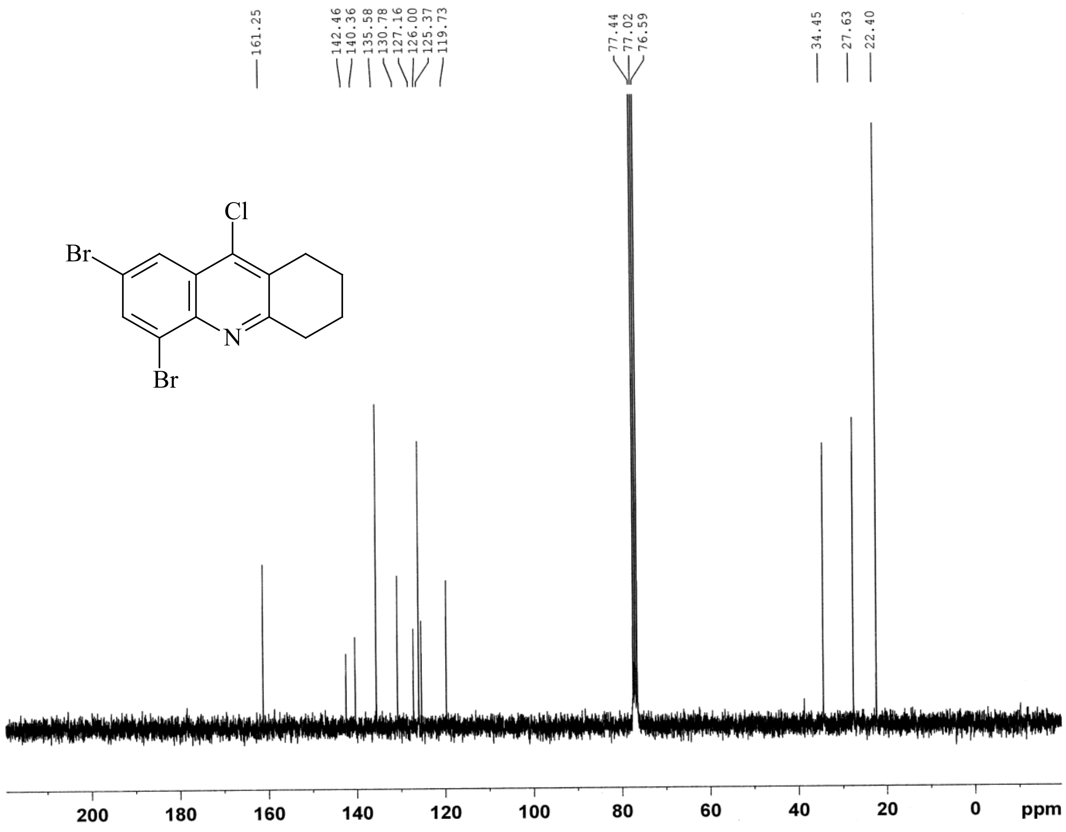

^{13}C NMR (75 MHz, $CDCl_3$) of 2,4-dibromo-9-chloro-5,6,7,8-tetrahydroacridine (2)

4. Experimental procedure for the Sonogashira coupling and spectroscopic data for 2,4-bis(2-arylethynyl)-9-chloro-5,6,7,8-tetrahydroacridine derivatives 4a–g:

2,4-Dibromo-9-chloro-5,6,7,8-tetrahydroacridine (**2**, 372.89 mg, 1 mmol, 1 equiv), arylacetylene (2.2 mmol, 2.2 equiv), $Pd(PPh_3)_4$ (6.93 mg, 0.006 mmol, 0.6 mol %) and CuI (2.285 mg, 0.012 mmol, 1.2 mol %) were added to a dried glass pressure tube. The tube was evacuated and backfilled three times with argon, then diisopropylethylamine (4.0 mL) was added. The tube was sealed with a Teflon cap and heated to 80 °C for 3–4 hours until completion of the reaction (reaction evolution was monitored by TLC). The mixture was then cooled to room temperature and the solvent was removed under reduced pressure. Water (10 mL) was added and the solution was extracted using CH_2Cl_2 (3 × 10 mL). The combined organic layers were dried (Na_2SO_4) and the solvent was evaporated under reduced pressure. The crude product was purified by column chromatography using heptanes/ethyl acetate 9:1 as eluent.

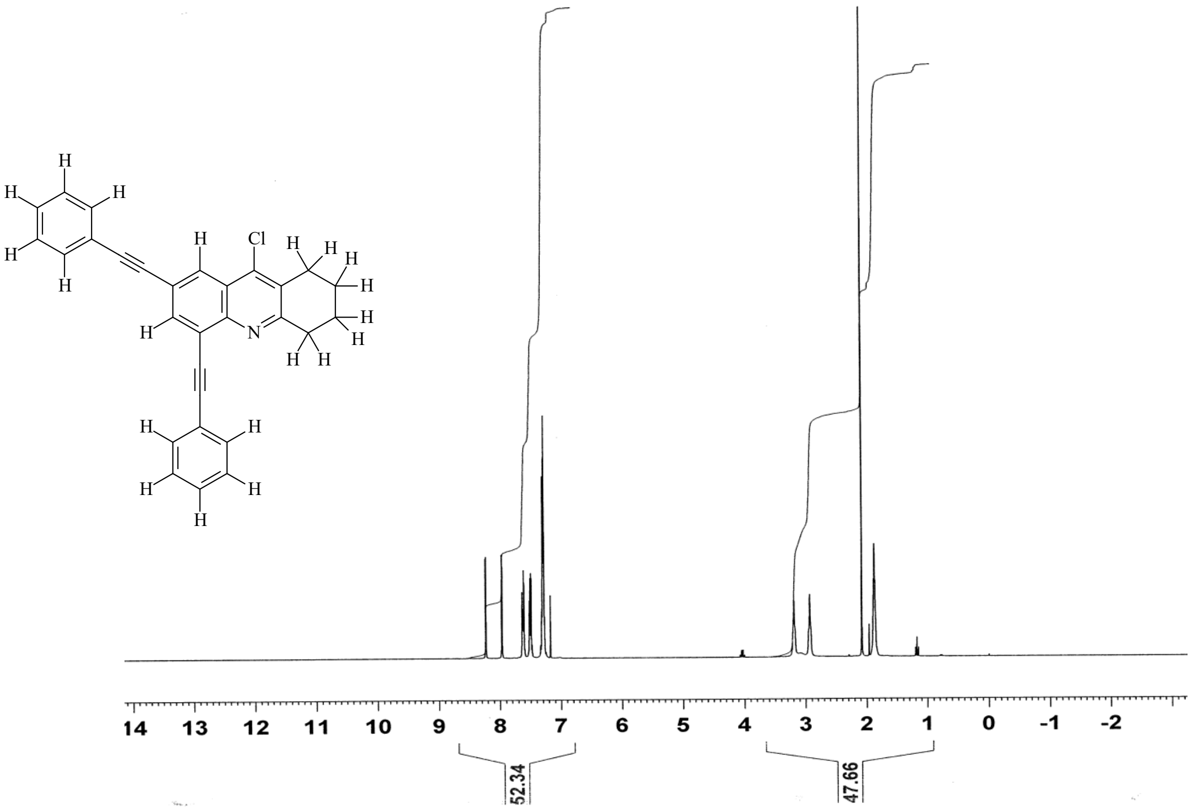

^{1}H NMR (300 MHz, $CDCl_3$) of 2,4-bis(2-phenylethynyl)- 9-chloro-5,6,7,8-tetrahydroacridine (4a)

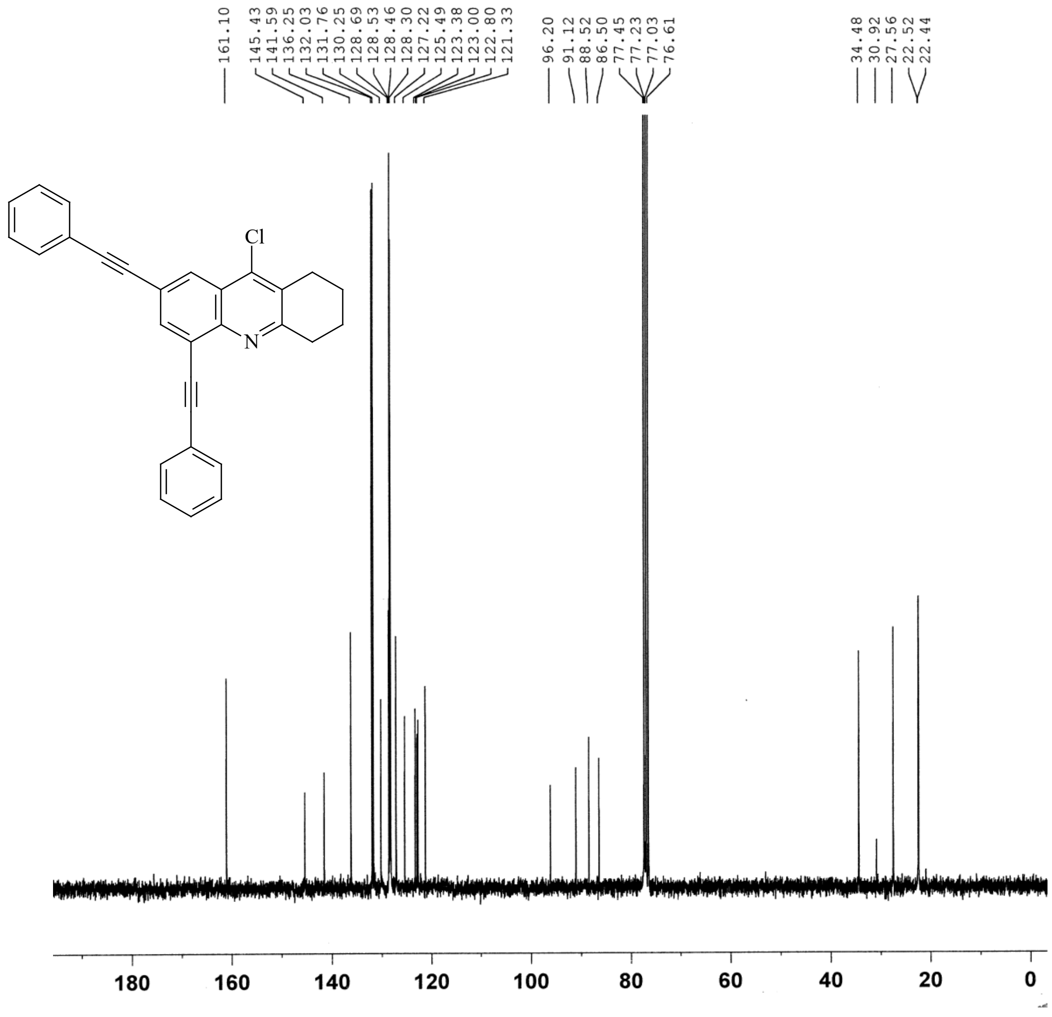

^{13}C NMR (75 MHz, $CDCl_3$) of 2,4-bis(2-phenylethynyl)- 9-chloro-5,6,7,8-tetrahydroacridine (4a)

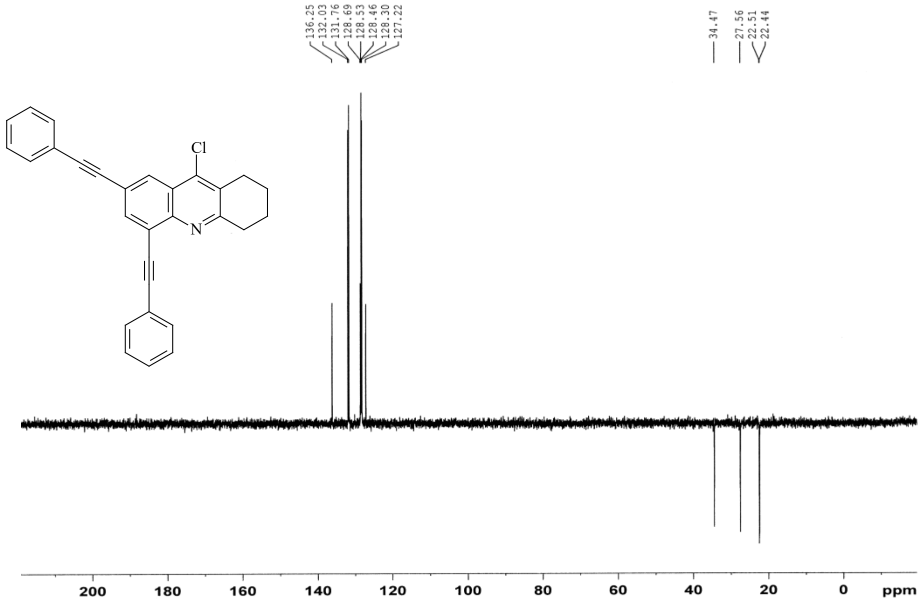

^{13}C NMR DEPT 135 spectrum of 2,4-bis(2-phenylethynyl)-9-chloro-5,6,7,8-tetrahydroacridine (4a)

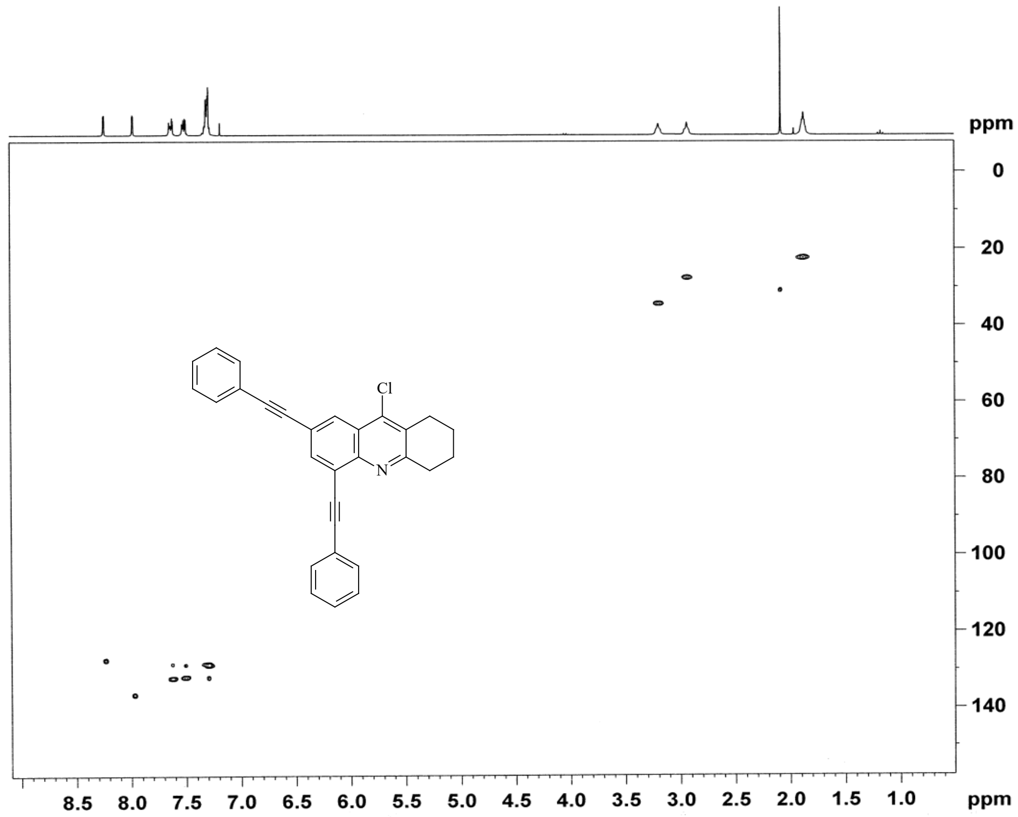

^{1}H,^{13}C– HSQC spectrum of 2,4-bis(2-phenylethynyl)-9-chloro-5,6,7,8-tetrahydroacridine (4a)

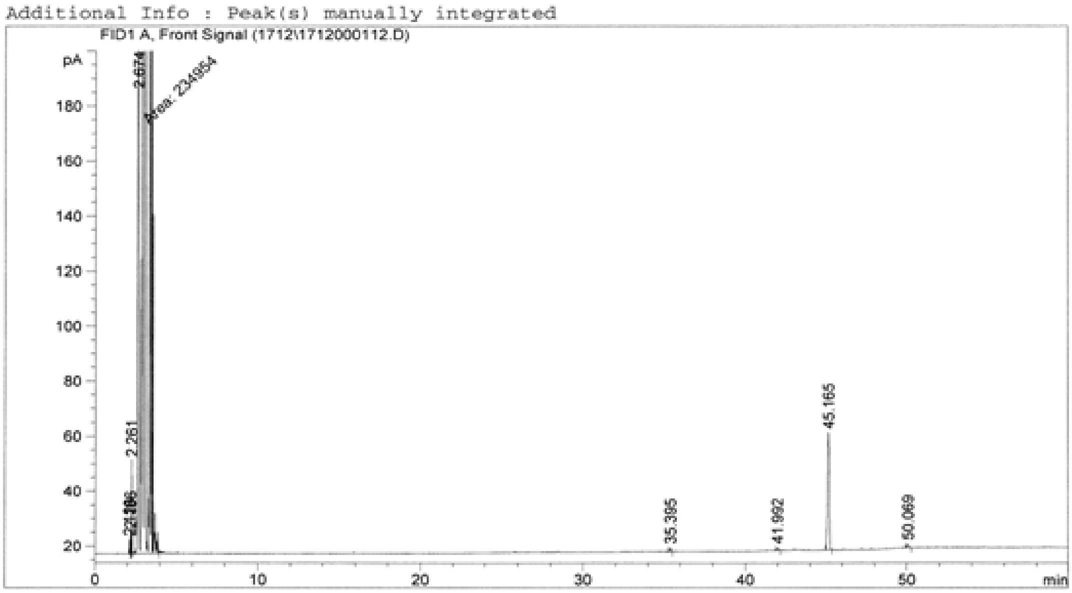

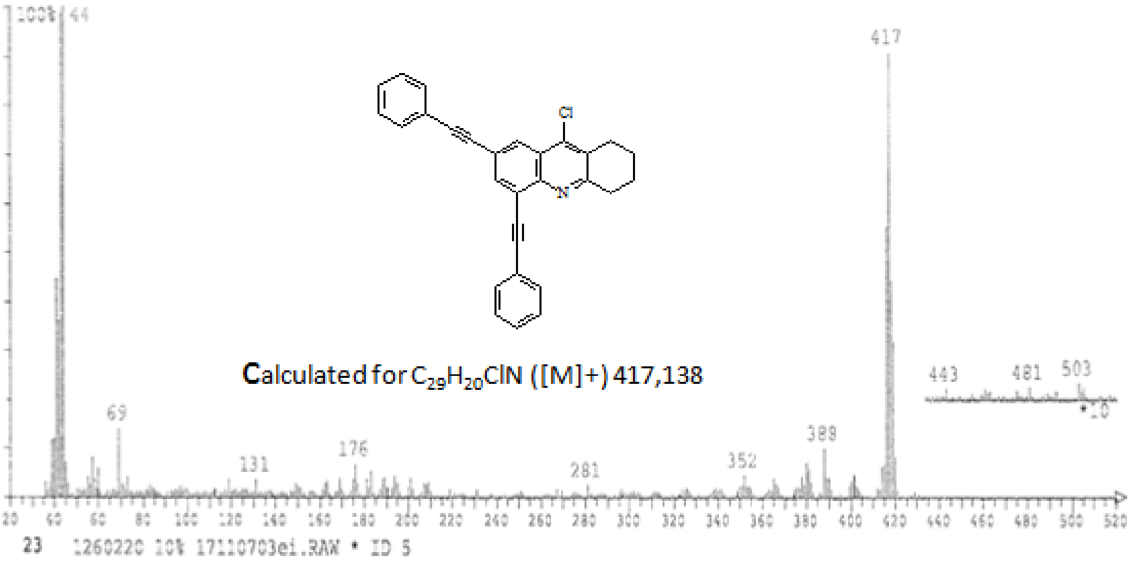

GPC and EIMS spectra of 2,4-bis(2-phenylethynyl)-9-chloro-5,6,7,8-tetrahydroacridine (4a)

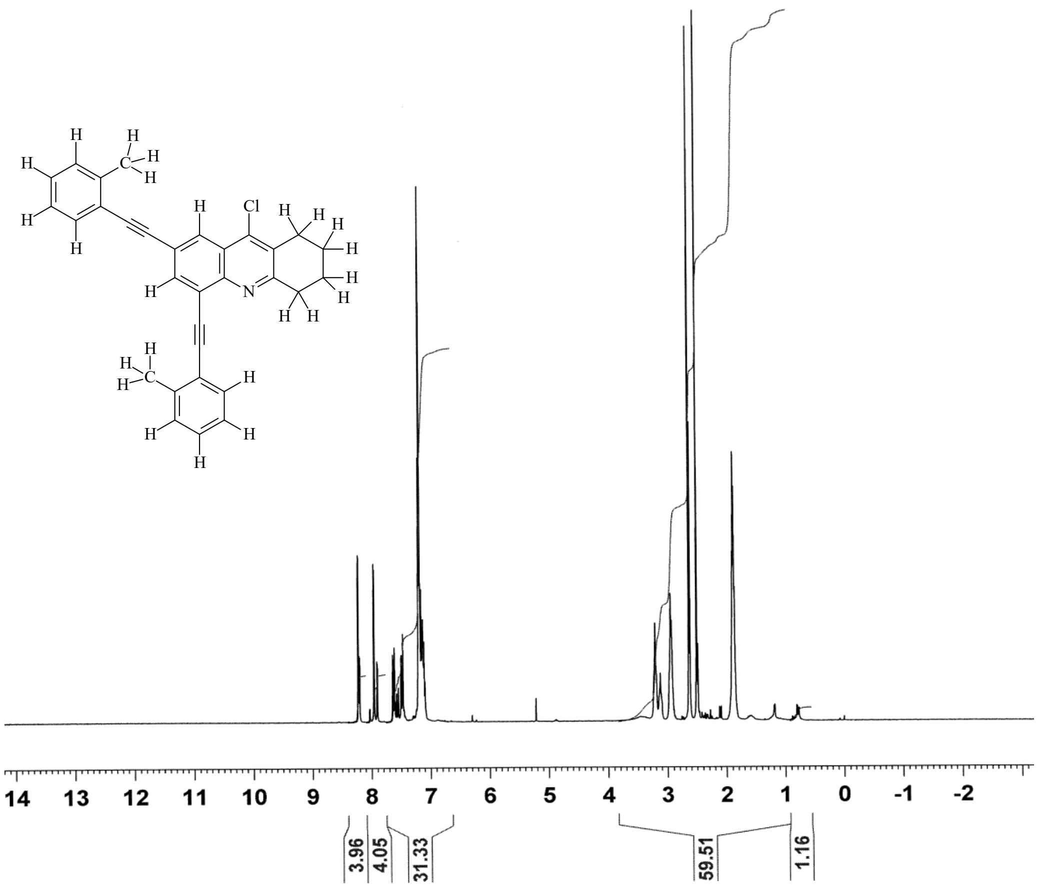

^{1}H NMR (300 MHz, CDCl$_3$) of 2,4-bis(*o*-tolylethynyl)-9-chloro-5,6,7,8-tetrahydroacridine (4b)

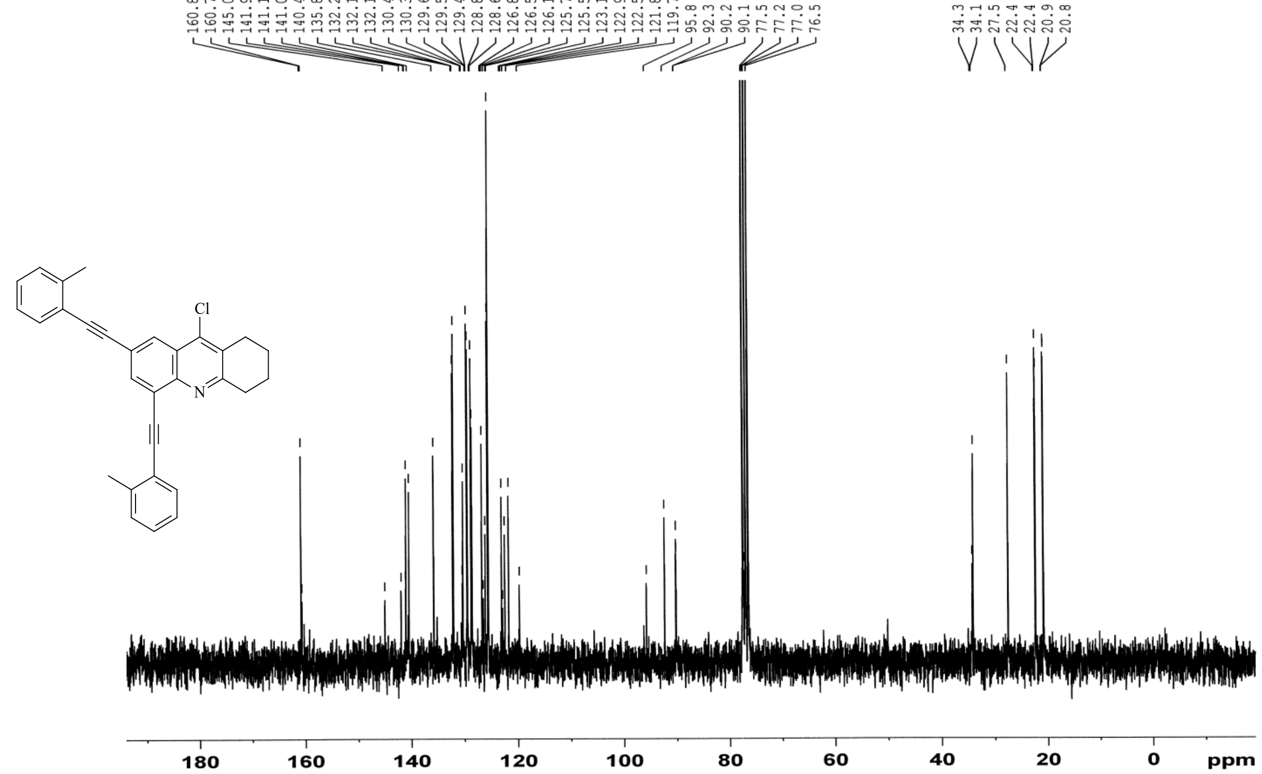

^{13}C NMR (75 MHz, $CDCl_3$) of 2,4-bis(*o*-tolylethynyl)-9-chloro-5,6,7,8-tetrahydroacridine (4b):

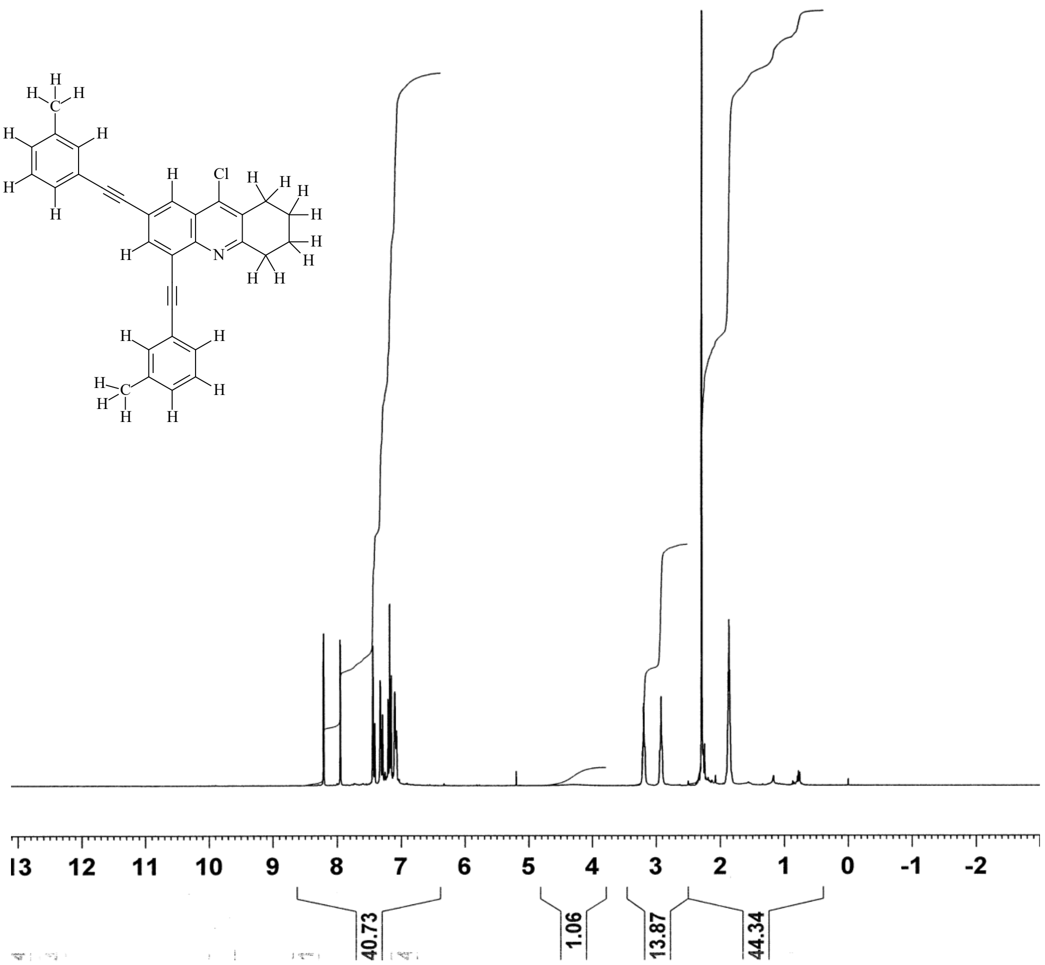

^{1}H NMR (300 MHz, CDCl$_3$) of 2,4-bis(*m*-tolylethynyl)-9-chloro-5,6,7,8-tetrahydroacridine (4c)

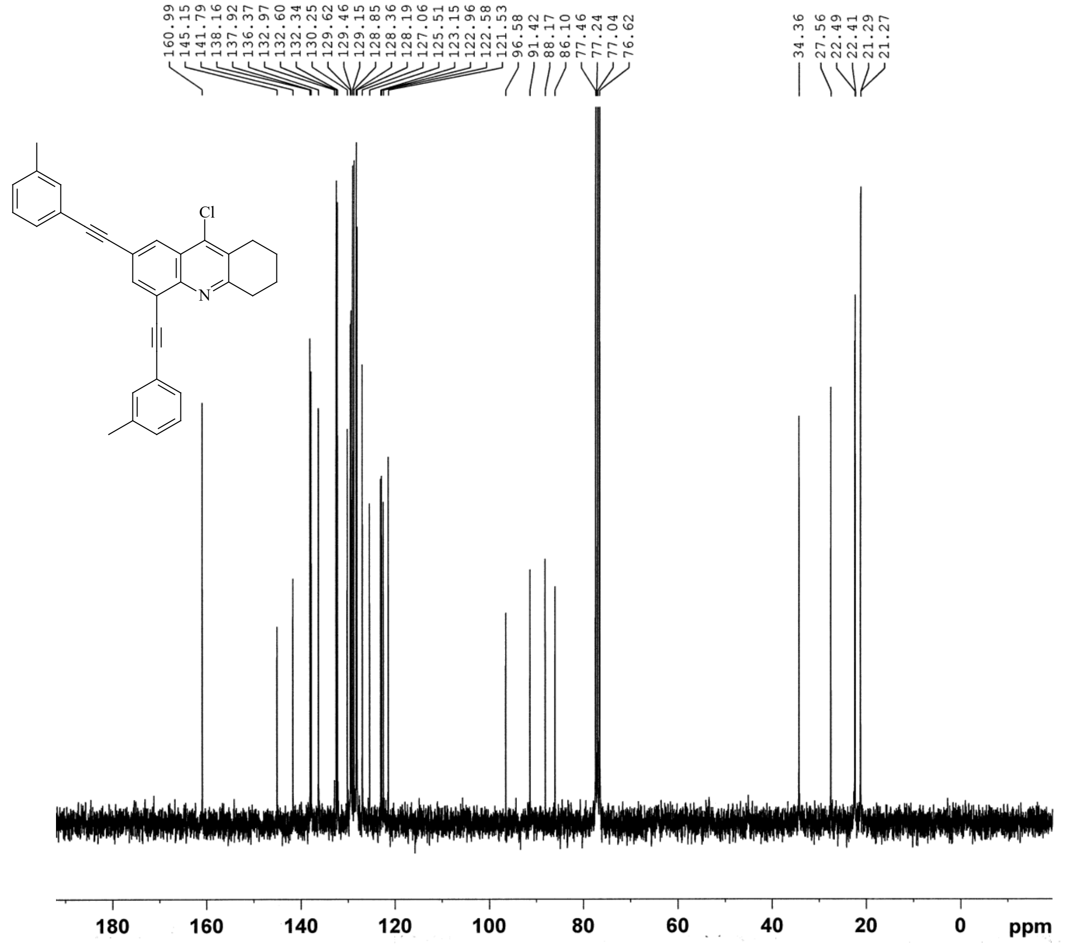

^{13}C NMR (75 MHz, CDCl*3*) of 2,4-bis(*m*-tolylethynyl)-9-chloro-5,6,7,8-tetrahydroacridine (4c)

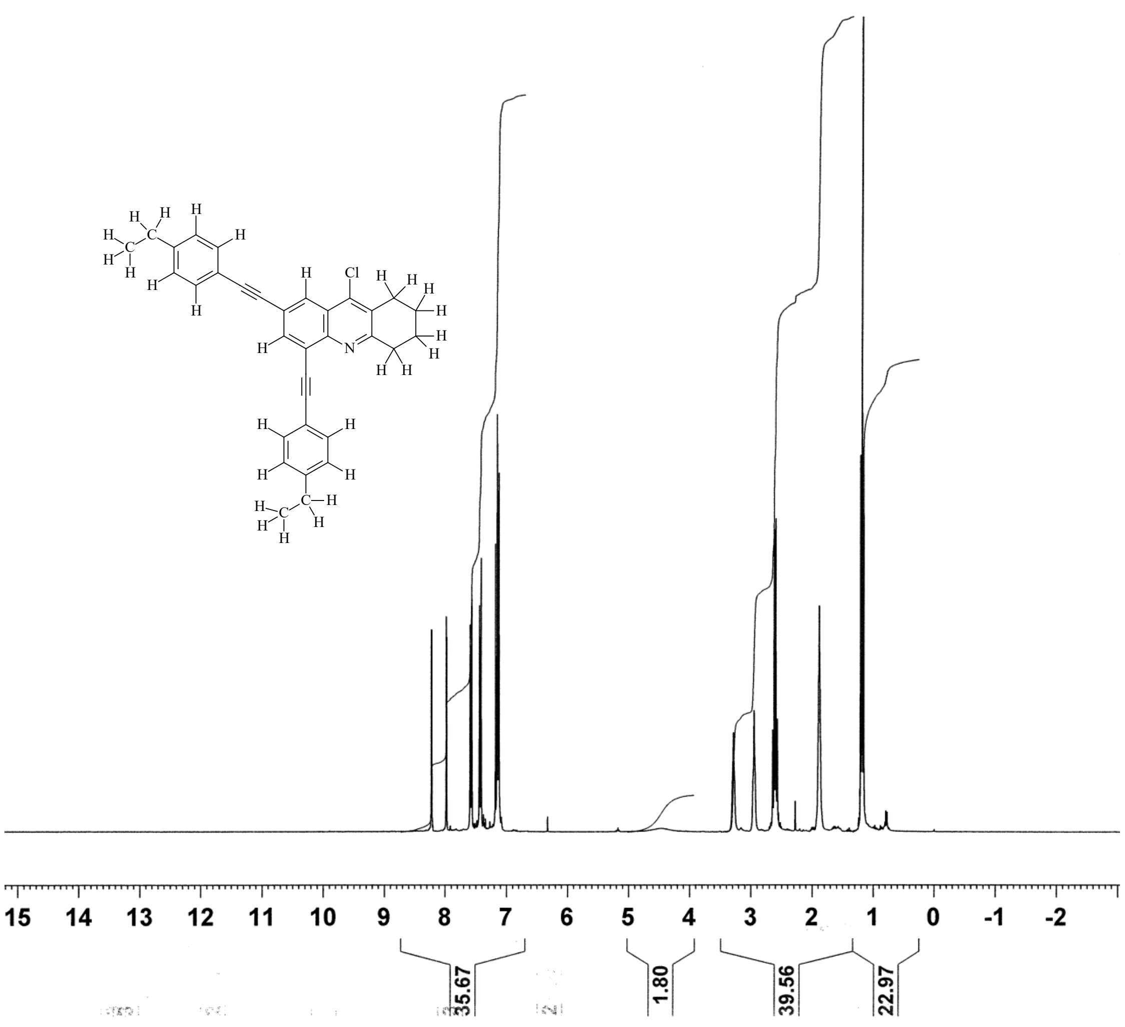

^{1}H NMR (300 MHz, $CDCl_3$) of 2,4-bis((*p*-ethylphenyl)ethynyl)-9-chloro-5,6,7,8-tetrahydroacridine (4d)

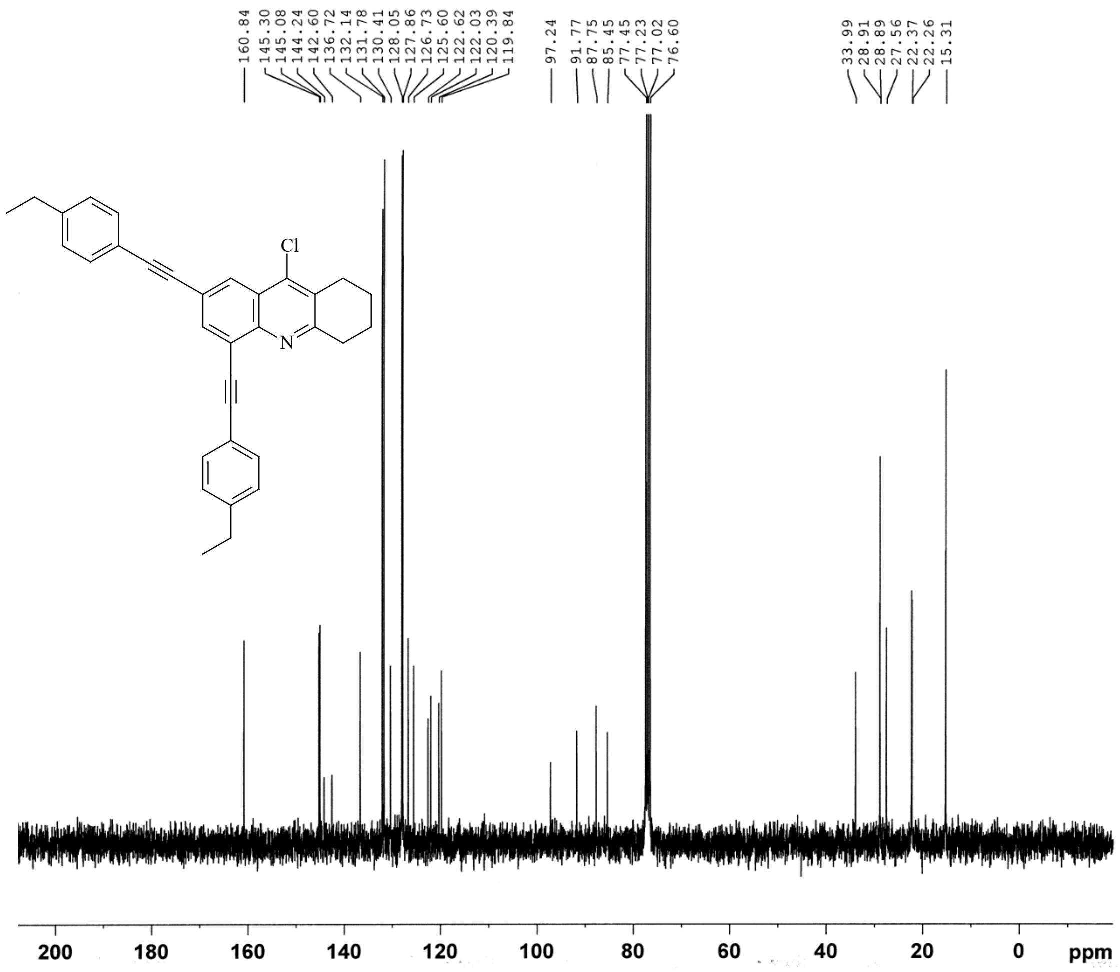

^{13}C NMR (75 MHz, $CDCl_3$) of 2,4-bis((*p*-ethylphenyl)ethynyl)-9-chloro-5,6,7,8-tetrahydroacridine (4d)

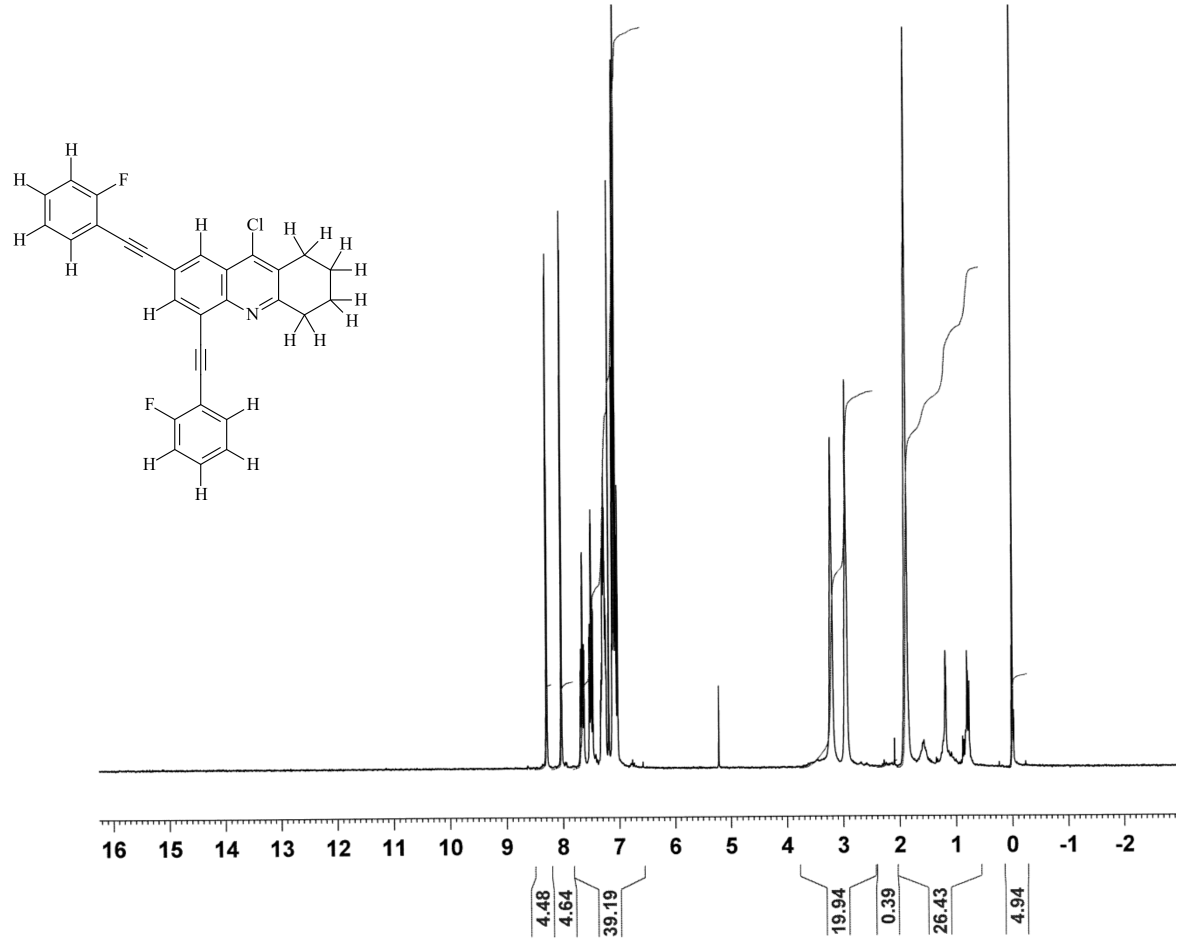

^{1}H NMR (300 MHz, $CDCl_3$) of 2,4-bis((*o*-fluorophenyl)ethynyl)-9-chloro-5,6,7,8-tetrahydroacridine (4e)

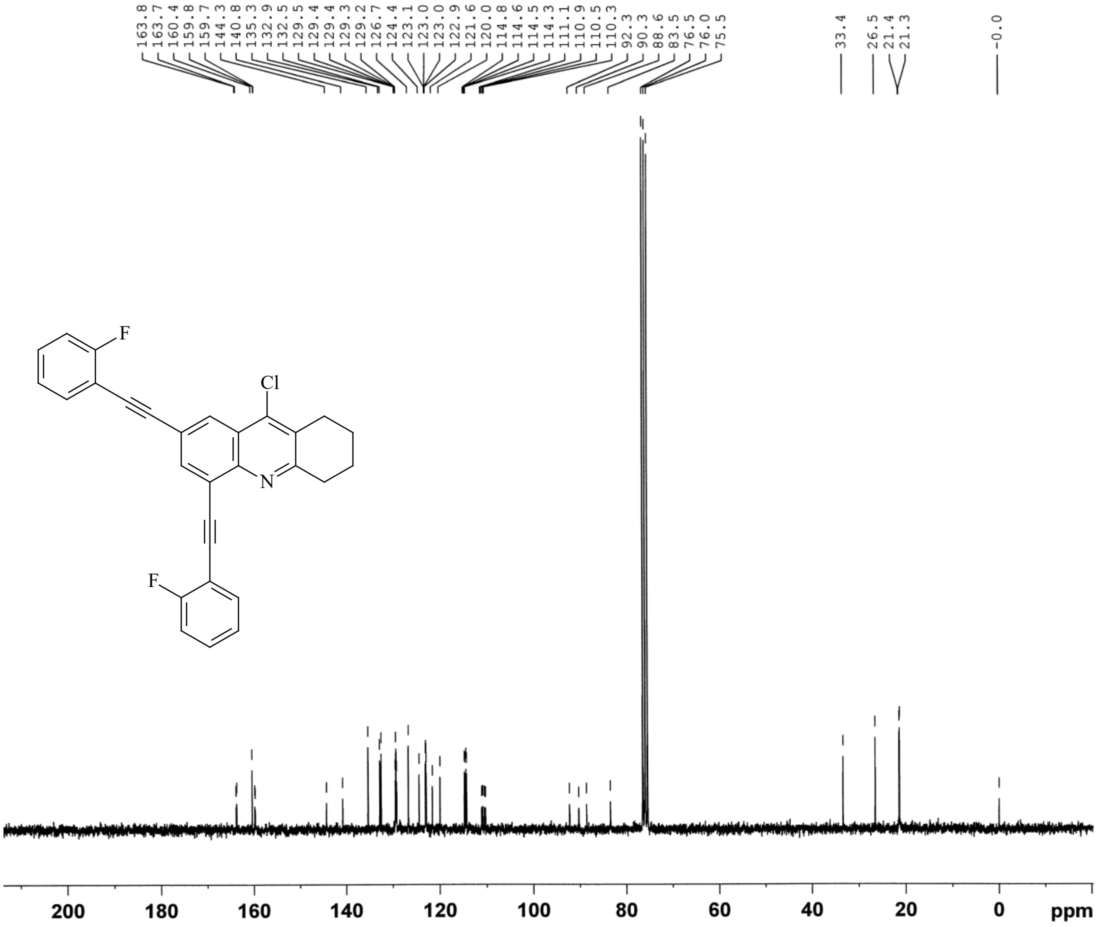

^{13}C NMR (75 MHz, $CDCl_3$) of 2,4-bis((*o*-fluorophenyl)ethynyl)-9-chloro-5,6,7,8-tetrahydroacridine (4e)

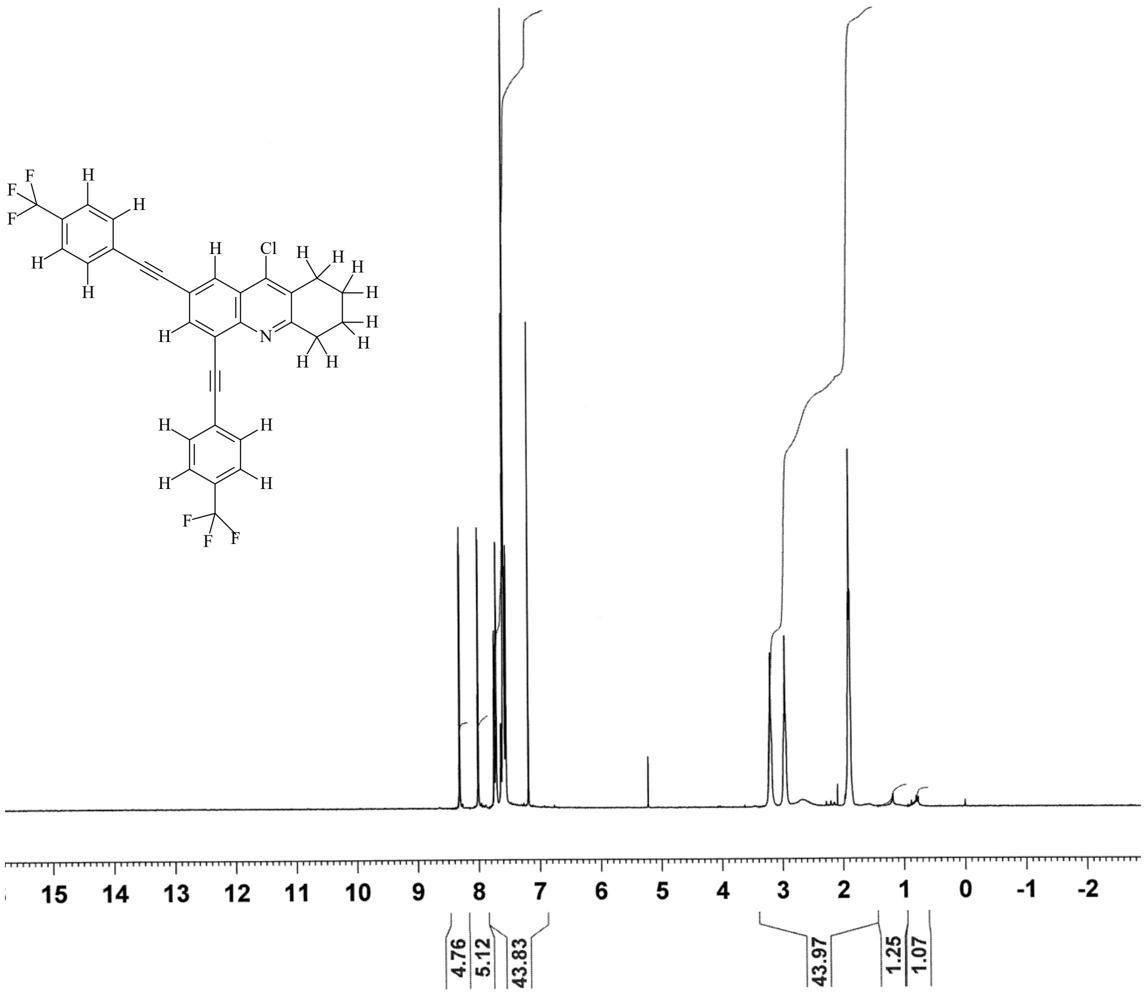

^{1}H NMR (300 MHz, CDCl$_3$) of 2,4-bis((*p*-trifluoromethylphenyl)ethynyl)-9-chloro-5,6,7,8-tetrahydroacridine(4f)

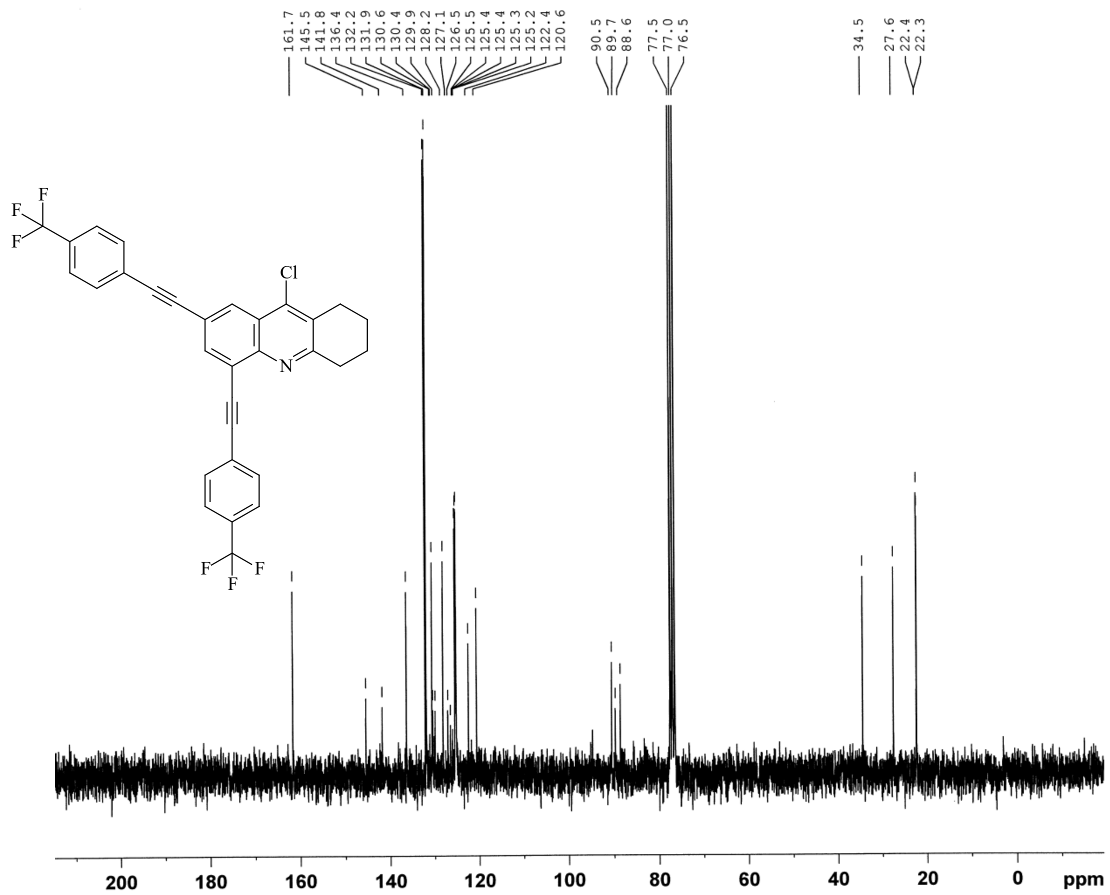

^{13}C NMR (75 MHz, $CDCl_3$) of 2,4-bis((*p*-trifluoromethylphenyl)ethynyl)-9-chloro-5,6,7,8-tetrahydroacridine(4f)

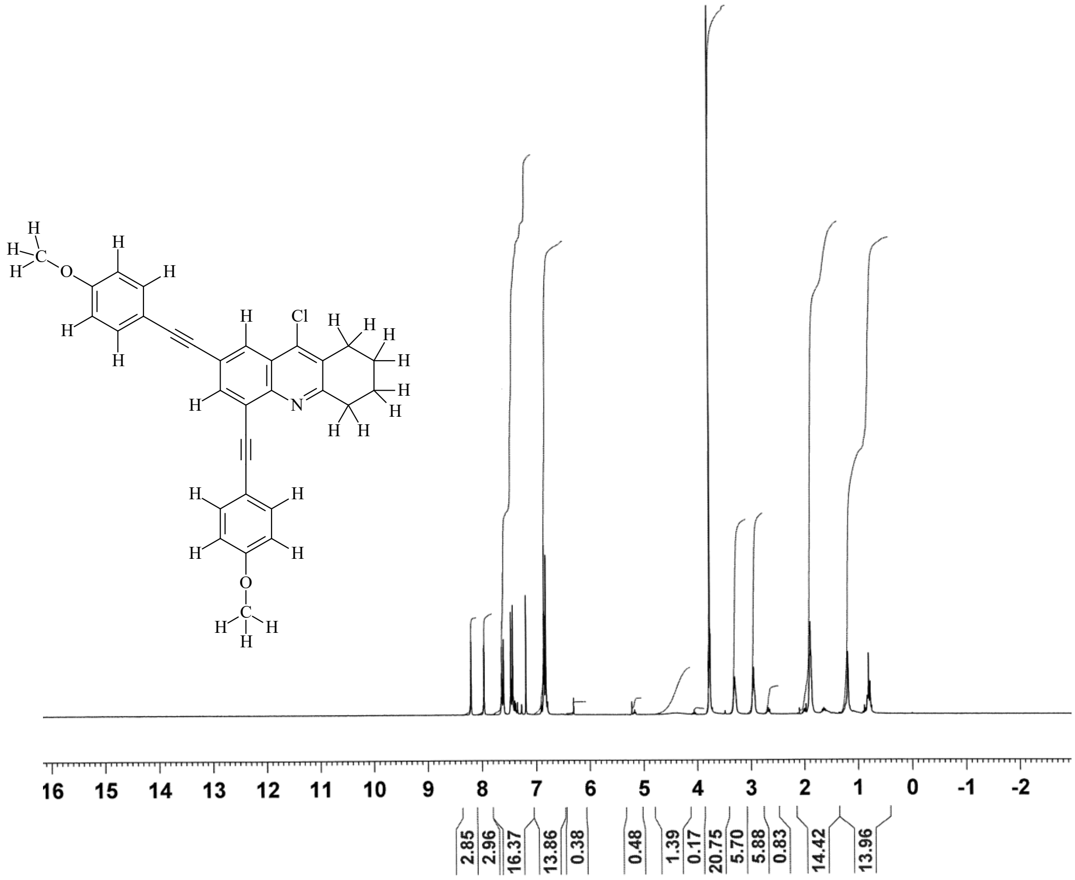

^{1}H NMR (300 MHz, $CDCl_3$) of 2,4-bis((*p*-methoxyphenyl)ethynyl)-9-chloro-5,6,7,8-tetrahydroacridine (4g)

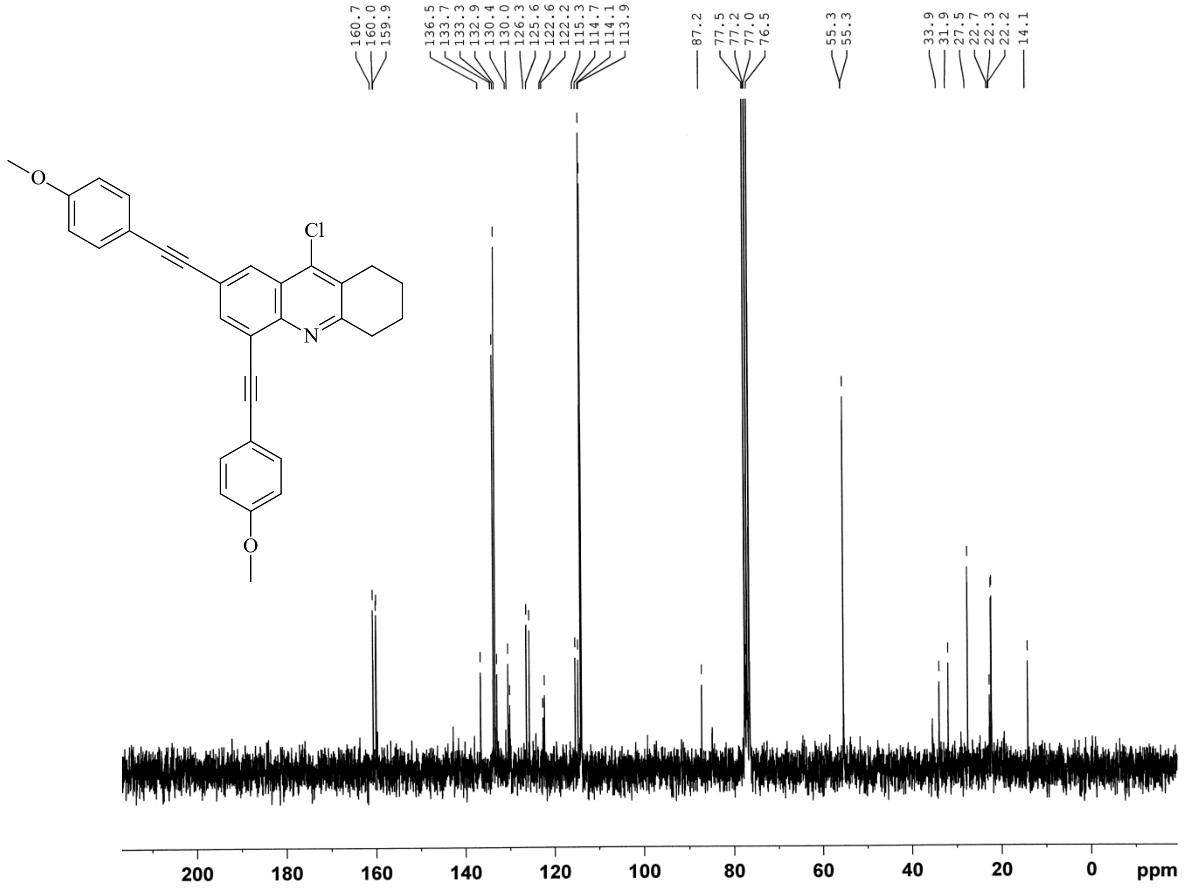

^{13}C NMR (75 MHz, $CDCl_3$) of 2,4-bis((*p*-methoxyphenyl)ethynyl)-9-chloro-5,6,7,8-tetrahydroacridine (4g)
